# Innate Multigene Family Memories Are Implicated in the Viral-Survivor Zebrafish Phenotype

**DOI:** 10.1371/journal.pone.0135483

**Published:** 2015-08-13

**Authors:** Amparo Estepa, Julio Coll

**Affiliations:** 1 Department of Biotechnology, Instituto Nacional Investigaciones Agrarias (INIA), Madrid, Spain; 2 Instituto de Biología Molecular y Celular, Universidad Miguel Hernández, Elche (UMH), Alicante, Spain; Purdue University, UNITED STATES

## Abstract

Since adaptive features such as memory were discovered in mammalian innate immunity, interest in the immunological status of primitive vertebrates after infections has grown. In this context, we used zebrafish (*Danio rerio*), a primitive vertebrate species suited to molecular and genetic studies to explore transcriptional memories of the immune system in long-term survivors of viral haemorrhagic septicemia virus infections. Immune-gene targeted microarrays designed in-house, multipath genes, gene set enrichment, and leading-edge analysis, reveal unexpected consistent correlations between the viral-survivor phenotype and several innate multigene families. Thus, here we describe in survivors of infections the upregulation of the multigene family of proteasome subunit macropains, zebrafish-specific novel gene sets, mitogen activated protein kinases, and epidermal growth factor. We also describe the downregulation of the multigene families of c-reactive proteins, myxovirus-induced proteins and novel immunoglobulin-type receptors. The strength of those immunological memories was reflected by the exceptional similarity of the transcriptional profiles of survivors before and after re-infection compared with primary infected fish. On the other hand, the high levels of neutralizing antibodies in the blood plasma of survivors contrasted with the depletion of transcripts specific for most cell types present in lymphoid organs. Therefore, long-term survivors maintained unexpected molecular/cellular memories of previous viral encounters by modulating the expression levels of innate multigene families as well as having specific adaptive antibodies. The implications of the so-called "trained immunity" for future research in this field are also discussed.

## Introduction

To date it has been difficult to assess the degree to which innate memories contribute to *in vivo* protection against viral infections. Innate memories may complement the well-known adaptive memory responses, such as viral-specific antibodies (Abs). Thus, adaptive features such as the innate memories typically found in invertebrates, are now being increasingly found in mammalians [1, 2, 3, 4, 5]. Given that fish are primitive vertebrates with an early immunological system (absence of IgG switch or IgM maturation, mucosal IgT/IgZ, phagocytic B-cells, etc), they might rely more on innate rather than adaptive immune responses to tackle viral diseases [6], however this notion has to be proven. In this regard, neutralizing Abs (NAbs) are not detected in some fish surviving a viral disease, such as occurs in salmonids with viral haemorrhagic septicemia virus (*VHSV*) [7–10]. Other mechanisms have been postulated to explain the viral resistance of these fish, including the participation of specific cytotoxic cells (CTL), *in vitro* non-neutralizing *in vivo* protecting antibodies, innate immunity memory, among others. However, such alternative mechanisms are not supported by enough experimental data. Zebrafish offer a suitable model to study the notion of innate immunity memories to viral diseases.

Typical adaptive features of innate immunity known as "trained immunity" are recently been reported in mammalians [1, 2, 3, 4, 5]. To date, the main features of such immunity are as follows: **i)** it confers protection by B-/T-cell-independent mechanisms; **ii)** it involves macrophage/natural killer (NK) cells [11–13]**; iii)** it remembers cross-protection to homologous and heterologous pathogens; **iv)** it enhances pathogen detection by means of several pattern recognition receptors and subsequent inflammatory responses [2, 14]; and **v)** it generates gene variation by epigenetic reprogramming (alternative splicing, DNA/histone modifications, miRNA, etc) rather than by genetic recombination [15]. Until now, there are only a few indirect lines of evidence of trained immunity in fish. For instance, a DNA vaccine against *VHSV* rhabdovirus also protects against nodaviruses [16], vaccination with G_*VHSV*_-derived peptides confers long-term protection against unrelated rhabdoviruses [17] and protection against bacterial pathogens can be induced in IgM knock-out (*rag1* ^*-/-*^) zebrafish mutants [18]. However, genes implicated in this trained immunity-like responses are unknown. Further clarification of this question will be fundamental to improve, design, follow up, and/or apply more efficacious vaccines and vaccination protocols in fish farming (i.e. wide pathogen-range vaccines).

Here we chose, the *VHSV* rhabdovirus and zebrafish (*Danio rerio*) as biological models. In this regard, *VHSV* has the greatest impact on salmonid farming worldwide [19, 20] and successful *VHSV* vaccination [21], microarray studies [22] and infection of larvae [23] and adults [21] had been described in zebrafish. Furthermore, protocols to reproducibly increase zebrafish resistance to *VHSV* have been developed in our laboratories [24]. To explore long-term memories to previous *VHSV* encounters, we compared the immunological responses of zebrafish phenotypes surviving *VHSV* vaccination and booster for months (VHSVS) with those of zebrafish phenotypes 2-days after *VHSV* infections, including primary (VHSV+) and after booster (VHSVS+) phenotypes.

Results published previously [22] and preliminary analysis of the present microarray data, showed limitations of the human-based pathway enrichment analysis using commercially available zebrafish microarrays when describing immunological changes in zebrafish after viral infections. As an alternative, we designed our own in-house microarray using oligo probes selected from human/zebrafish orthologous immune-related KEGG/WIKI pathways and zebrafish mRNAs selected by keyword searches in Gene Banks. The immune-targeted microarray resulted in a 3- to 4-fold enrichment in immune-related genes. In addition, the hybridization data were analyzed not only by using gene-to-gene (i.e. modulated MultiPathway Genes, mMPG) [25] but also gene set enrichment analysis (GSEA) [26] methods. Among other findings, we discovered previously unknown contributions of several innate multigene families (groups of genes encoding proteins with similar sequences) to the VHSVS phenotype. These families included, upregulated proteasome subunit macropain proteins (*psm*) and downregulated c-reactive protein (*crp*), myxovirus-induced protein (*mx*), and novel immunoglobulin-type receptors (*nitr*). As a result of their long-term memory to previous exposures to *VHSV* and gene polymorphisms, all these multigene families are candidates for trained immunity phenomena. Furthermore, in addition to identifying the preferential participation of some human-like zebrafish pathways already described, hypothetical gene sets (GSs) consisting of genes whose expressions were apparently coordinated in VHSVS, pointed to the existence of novel fish networks that better explained this phenotype. Moreover, surprisingly, while high levels of NAbs were present in VHSVS plasma, the lymphoid organs were not only depleted in B cells (IgM+ cells), but also in Treg, Th1, Th2, and dendritic cells. This observation would suggest that cells migrate to the entry point in peripheral tissues. All of the above findings indicate that vaccinated plus booster VHSVS zebrafish maintain a high level of innate multigene families (i.e. *psm*) and cellular changes in order to resist *VHSV* infections, in addition to maintaining adaptive responses (mainly represented by NAbs). Those protective mechanisms were so strong that few additional transcriptional changes could be detected after VHSVS re-infection in the VHSVS+ phenotype. These findings also point to new lines of research into the newly described associations of the multigene families in fish with trained immunity described in mammals [3, 11, 12, 27].

## Materials and Methods

### Viral haemorrhagic septicemia virus (*VHSV*)

The viral haemorrhagic septicemia virus (*VHSV*) strain 07.71 (accession number AJ233396) isolated from rainbow trout *Oncorhynchus mykiss* [28] was replicated in cells from the fathead minnow fish (*Pimephales promelas*) (ATCC, Manassas, Vi, USA), called EPC. Cells were grown at 28°C with 5% CO_2_ in RPMI Dutch modified 20 mM HEPES cell culture medium supplemented with 10% fetal calf serum (FCS), 1 mM piruvate, 2 mM glutamine, 50 μg/ml gentamicin and 2.5 μg/ml fungizone (Sigma, St.Louis, Missouri, USA). Supernatants from *VHSV*-infected EPC cell monolayers (2% FCS, 10 mM Tris pH 8.0, no CO_2_) were cleared by centrifugation and kept at -70°C until used for *in vivo* experiments. To obtain concentrated *VHSV* for neutralizing assays, supernatants were centrifuged at 60.000 *g* for 180 min at 4°C, and pellets were frozen at -70°C until use. *VHSV* was titrated by the focus forming units (ffu) assay [29].

### Zebrafish (*Danio rerio*)

#### Adult naïve zebrafish

Adult zebrafish weighting 700–900 mg (3–4 cm in length) were obtained from a local pet shop (Aquarium Madrid, Madrid, Spain). They were maintained at 24–26°C in 30 l aquaria provided with biological filters, and fed a commercial diet.

#### Generation of primary infected VHSV+ phenotype

Zebrafish (n = 15–35 per experiment) were moved to 2 liter mini-aquaria maintained at 14°C, and equipped with biological filters. After acclimation for 7 days, groups of 10 zebrafish were infected-by-immersion in 10^7^ focus-forming units (ffu) of *VHSV* per ml for 2 h in 50 ml of water at pH 8 [30] and then returned to their mini-aquaria. In parallel, non-infected (NI) zebrafish were mock-infected with cell culture medium. Fish were euthanized 2-days after infection and plasma and lymphoid organs were harvested (see below). Alternatively, mortality (see later for endpoint details) was recorded over 1 month at 14°C (S1 Fig).

#### Generation of vaccinated plus booster VHSVS phenotype

To obtain enough zebrafish surviving *VHSV* infection, we followed similar procedures to those previously described [24, 31]. Briefly, fish were anesthetized (see below) and then intraperitoneally vaccinated by injection of 10^6^ ffu of *VHSV* in 10μl of PBS (phosphate-buffered saline) and maintained at 18°C (higher than the optimal temperature for *VHSV* replication). Vaccination by intraperitoneal injection at 18°C was more reproducible than earlier attempts made by immersion (not shown). After 1-month at 18°C, zebrafish were maintained for 2-months at 24–26°C. Three-months after vaccination, survivors were then infected-by-immersion (booster) at 14°C as described above for VHSV+. They were then maintained for 1-month at 14°C and then for 2-months at 24–26°C. Six-months after vaccination, plasma and lymphoid organs were harvested from euthanized survivors of vaccination plus booster (VHSVS) (S1 Fig).

#### Generation of infected after booster VHSVS+ phenotype

VHSVS were re-infected-by-immersion at 14°C with *VHSV*. Two-days later, plasma and lymphoid organs were harvested from euthanized fish. Alternatively, VHSVS+ were maintained at 14°C for 1-month to record mortality (S1 Fig).

#### Generation of zebrafish surviving a natural infection with bacteria

Zebrafish showing a daily mortality rate of 1–2% during the summer months at 24–26°C, maintained a chronic level of natural bacterial infection characterized by the presence of 2.3 x 10^6^ bacteria per 10^6^ head kidney/spleen cells. Infecting bacteria were identified as *Aeromonas hydrophila* and *Vibrio fluvialis* (Microbiological Service of the Fundación Hospital Alarcon, Madrid Spain). Bacterial survivors were used for experiments 5 months after the first deaths were detected.

#### Harvesting blood plasma from zebrafish

Anesthetized zebrafish (see below) were bled by cutting the final end of the tails. The blood from each individual fish was collected in 200 μl of sterilized anticoagulant media (0.64 g sodium citrate, 0.15 g EDTA, 0.9 g sodium chloride per 100 ml of water) and immediately centrifuged at 1000 g for 3 min to obtain plasma. To prevent individual complement interferences with the *VHSV*-neutralization microassay [29], plasma was de-complemented by heating to 45°C for 30 min and then kept frozen at -20°C until use.

#### Harvesting lymphoid organs from zebrafish

For each biological replica, head kidney and spleens (lymphoid organs) were harvested and pooled from 3 euthanized zebrafish (see below). Each replica was kept in RNAlater (Qiagen) and 4 replicas were made per phenotype. RNAs from pooled lymphoid organs were extracted (Qiagen) and kept frozen at -80°C until all the replicas were hybridized to microarrays and processed simultaneously.

#### Zebrafish handling

During the survival studies or to record mortality from 2 to 30 days, the *VHSV*-infected fish were monitored 2–4 times a day to minimize suffering. Those fish showing external haemorrhages and/or abnormal swimming behavior (endpoint criteria) were euthanized by submersion in ice water (5 parts ice/1 part water, 0–4°C) for 10 min and then exposed to an overdose of methanesulfonate 3-aminobenzoic acid ethyl ester (MS222, 300 mg/l) for > 10 min after cessation of opercular movement, as recommended by the “Guidelines for Use of Zebrafish in the NIH Intramural Research Program” (http://oacu.od.nih.gov/ARAC/documents/Zebrafish.pdf). Fish were anesthetized with MS222 at 90 mg/l to obtain blood, which was harvested as described above and euthanized by an overdose of MS222 to extract lymphoid organs. There were no unexpected deaths other than those caused by *VHSV* infection as determined by RTqPCR of the N nucleoprotein mRNA of *VHSV* (N_*VHSV*_)(see details later).

#### Ethics statement

Zebrafish were handled in accordance with the National and European guidelines on laboratory animal care. The fish protocols were approved by the Ethics Committee of the Instituto Nacional de Investigaciones Agrarias (authorization CEEA 2011/022), following the specific national guidelines for type III experimentation, as stipulated in Annex X of permission RD53/2013.

### Microarray design and analysis

#### Design of zebrafish immune-targeted microarray

To re-design a first ID41401 version of immune-targeted in-house zebrafish microarray, new probes from selected KEGG and WIKI pathways and from keywords were added [25]. Thus, we selected immune-relevant human (*Homo sapiens*, hsa) 32 pathways from the Kyoto Encyclopedia of Genes and Genomes (KEGG) (http://www.genome.ad.jp/kegg/) and 30 from the WIKI pathway data bases (accessed in February-March of 2013). Orthologous human/zebrafish mRNA symbols (*italics*) were searched and retrieved from each human KEGG pathway box gene in http://www.kegg.jp/ssdb-bin/ssdb_best?org_gene=hsa and http://www.genome.jp/dbget-bin/www_bget?dre, to obtain the corresponding zebrafish accession numbers in http://www.genome.jp/dbget-bin/get_linkdb?-t+10+dre, following previously described methods [25]. Zebrafish accession numbers were extracted from the WIKI pathways at pathways http://www.wikipathways.org/index.php?title=Special%3ABrowsePathways&browse=Danio_rerio. We also included 25 immune-related genes retrieved using keywords from the GenBank data base of zebrafish mRNAs (http://www.ncbi.nlm.nih.gov/) accessed in March 2012. Retrieved sequences were filtered for duplicates and non-related genes were eliminated manually. The S2 Fig shows a VENN diagram comparison of unique accession numbers of our new immune-targeted microarray version with a current commercial version of non-targeted microarray.

Oligo probes of 60-mer and melting temperature of 80 ± 3°C were then designed for each of the sequences using the Array Designer 4.3 program (Premier Biosoft Palo Alto CA, USA) and the zebrafish mRNA GenBank data base (accessed in April, 2013). To corroborate gene identification, the new design was first validated *in silico* using BLAST of an statistically significant number of probes. Our previous ID41401 platform version included in the new ID47562 platform version used for these experiments, was validated by RTqPCR in a previous study [25]. Twenty of the new probes of the ID47562 platform version were also validated by RTqPCR in the present work (see below), as previously described [25]. Finally, the list of 60-mer oligo probes in an 8x15K format was submitted to Agilent's microarray design tool (https://earray.chem.agilent.com/earray/search.do?search¼arrayDesign) and deposited in Gene Expression Omnibus GEO's GPL17670 (see S1 Table for a summary).

#### Transcript quantification after hybridization to the in-house immune-targeted microarray

Labeling of 2 μg of high quality RNA (50 μg/ml) and hybridization to the microarrays were performed by Nimgenetics (Cantoblanco, Madrid, Spain), complying with the Minimum Information About a Microarray Experiment (MIAME) standards as described in detail before [22, 25]. Raw and normalized data were deposited in the GEO bank at http://www.ncbi.nlm.nih.gov/geo/query/acc.cgi?acc, VHSV+ at GSE58823 and VHSVS/VHSVS+ at GSE57952.

### Identification of modulated MultiPath Genes (mMPG)

Normalizations using the sum of all probe fluorescence values for each microarray were performed as described before using the Origin pro vs8.6 program (Northampton, USA) [22, 25]. NI outliers (values outside means ± standard deviations) were masked from mean calculations (n = 4). Folds were then calculated by applying the formula, experimental normalized gene fluorescence value for each replica / NI mean. Fold outliers were then eliminated and their mean and standard deviations were calculated. Folds were obtained by comparing the following zebrafish phenotypes: VHSV+ versus NI, VHSVS versus NI and VHSVS+ versus NI. There were 154 MultiPath Genes (MPG), defined as those genes present in >6 pathways in the in-house microarray (S1 Table). Those MPG with fold means >2 or <0.5 significant at the p>0.05 level (n = 4) using the 2-tail independent t-test at p<0.05, were considered modulated (mMPG).

#### Gene Set Enrichment Analysis

To perform the Gene Set Enrichment Analysis (GSEA) (http://www.broad.mit.edu/GSEA) [26, 32], the 14541 gene probes of the in-house microarray and their fluorescent values were first downsized to 2274 unique genes and values (average of 6.4 probes per gene). The list of unique genes and values was ranked by the t-test statistic metric [26, 32] (similar results were obtained using the Signal-to-Noise ratio statistic). The ranked list was used to calculate Enrichment Scores (ES) by comparing the following zebrafish phenotypes: VHSV+ versus NI, VHSVS versus NI and VHSVS+ versus NI. As input GSs, we first used the human 10.295 GSs included in the GSEA web and then the 87 human/zebrafish orthologous pathway GSs from the in-house-designed microarray (S1 Table). The GSEA calculated individual gene enrichment scores (ES), overall ES for each GS and finally normalized ES (NES) to correct for the number of genes present in each GS. As suggested by GSEA, the most stringent cut-off value of <0.05 False Discovery Rate (FDR) was used for NES significance. The FDR method was chosen because only FDR corrected for both gene size and multiple hypothesis (null distribution from 1000 random gene combinations per GS). Because the zebrafish GS were derived from human/zebrafish orthologous pathways and that might be inaccurate, the Leading Edge Gene Analysis (LEGA) was used to search for empirically clustered genes (GS/LEGA matrixes) indicative of novel fish GSs.

### Validation by reverse transcriptase and quantitative polymerase chain reaction (RTqPCR)

Microarray results were validated by RTqPCR of selected genes by following the same procedures reported previously [22, 25]. The list contains 20 differentially expressed genes belonging to either multigene families (*crp*, *mx*) or mMPG (S2 Table) and the *ef1a* normalizer gene [33]. In addition, *VHSV* replication was estimated by measuring its N nucleoprotein (N_*VHSV*_) transcript levels. Forward and reverse primers amplifying 100–120 bp were designed using the Array Designer 4.3 program (Premier Biosoft Palo Alto CA, USA) (S2 Table). RNA from lymphoid organs was converted to cDNA (PrimeScript RT reagent kit, Takara, Japan) by following manufacturer instructions. The resulting cDNA (25 ng cDNA per sample) was mixed with Power SYBR green PCR Master Mix (Applied Biosystems) and amplified in a LineGene 9600 Real-Time PCR system (Bioer Technology Co, Bingjiang, China). The relative number of molecules were calculated from the cycle threshold (Ct) data using the 2^-delta^ relative quantitation method and normalized for each experiment using the *rplp0* gene [33]. Outliers (values > or < means ± standard deviations) were eliminated and the fold for each gene calculated by the formula: relative number of molecules from phenotype zebrafish / mean of relative number of molecules from NI. Means and standard deviations were then calculated (n = 4 biological replicas).

### Anti-*VHSV* neutralizing antibodies in zebrafish plasma

The high throughput method using EPC cell monolayers plated onto poly-D-Lys coated wells (Corning, New York, NY, USA) was used [24]. Briefly, de-complemented zebrafish plasma was pre-incubated with 300 ffu of purified *VHSV* per well. Then, *VHSV*-infected monolayers were incubated overnight, fixed with formaldehyde, permeabilized and stained with anti-N_*VHSV*_ MAb 2C9 [34]. EPC cell suspensions were obtained by trypsin digestion and analyzed in a BD FACS Canto II apparatus (Beckton Dickinson, San Agustin de Guadalix, Madrid, Spain) provided with a high throughput sampler (HTS). The number of fluorescent cells (*VHSV*-infected cells) over a threshold containing 95% (mean + 2 standard deviations) of non-infected EPC cells was then determined. The percentage of infected cells was calculated using the formula: 100 x number of cells with fluorescences above the threshold / total number of cells gated per well. *VHSV*-infected cell controls in the absence of added zebrafish plasma showed that 55% of the EPC cells were infected. The results were then expressed in % of neutralization by the formula: 100–100 x percentage of infected cells / 55.

### Flow cytometry profile of IgM^+^ lymphocytes

To obtain a MAb crossreacting with zebrafish IgM, a collection of 25 anti-trout IgM MAbs [35–38] was screened by flow cytometry for recognition of the lymphocyte population previously defined in zebrafish head kidney [39]. MAb 6B7 recognized the zebrafish lymphocyte population and therefore was used for these studies. Cells from lymphoid organs were pooled from 3 zebrafish for each phenotype and IgM staining was performed by slight modifications of the procedure described before for monolayers of cell lines [24]. Briefly, 150000 zebrafish lymphoid cells per well sedimented for 20 min were fixed to poly-D-Lys wells with 10% formaldehyde in PBS for 20 min. Then they were reversibly permeabilized with 0.05% Saponin, 0.01% N_3_Na in PBS for 15 min and stained with no MAb, an irrelevant MAb, FITC-phytohemaglutinin PHA (Vector, Barcelona, Spain) and anti-trout IgM MAb 6B7 and rabbit FITC-labeled anti-mouse IgG (Nordic, Tilburg, The Netherlands). The trypsinized cells were separated in 5 subpopulations by FSC and SSC profiles using the BD FACS Canto II apparatus. The number of fluorescent cells in each population over a threshold containing 95% (mean + 2 standard deviations) of cells stained by an irrelevant MAb was then determined in 10000 events per well. The percentage of cells in each population was calculated by the formula: number of cells in each population / total number of cells gated. The percentage of fluorescent cells in each cell population was calculated by the formula: 100 x number of cells with fluorescence above the threshold / total number of cells gated. Mean and standard deviations were then calculated (n = 2 determinations). Comparison of significance of the values was performed by using the t-test at the p<0.05 level.

## Results and Discussion

### Properties of *VHSV*-infected zebrafish phenotypes (VHSV+, VHSVS, VHSVS+)

**Fig 1:**
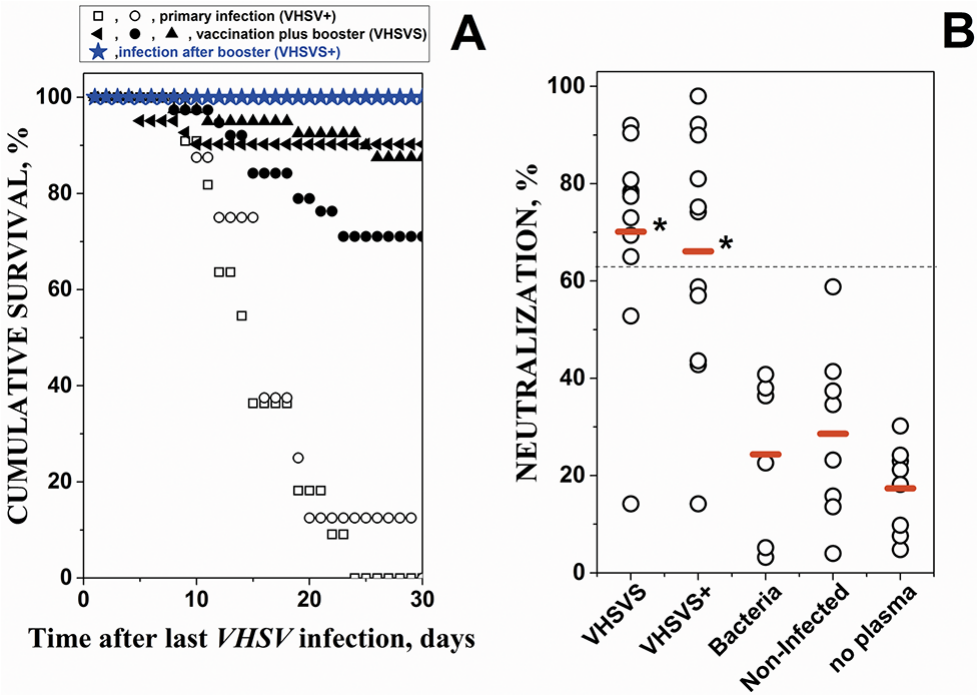
Cumulative percent survival of zebrafish after *VHSV* infections (A) and levels of anti-*VHSV* neutralizing antibodies (NAbs) in plasma (B). **A)** primary infected VHSV+ and vaccinated plus booster VHSVS were obtained as described in detail in methods and summarized in S1 Fig After infection-by-immersion in 10^7^ ffu of *VHSV* per ml, daily cumulative survival at 14°C was calculated for each experiment using the formula, 100 x (1- daily cumulative mortality / total mortality after 30 days) (n = 15 to 35 zebrafish per experiment). Different symbols (circles, squares, triangles and stars) correspond to independent experiments. **Open circles and squares**, primary infection-by-immersion at 14°C (VHSV+). **Solid symbols**, vaccination- by-injection at 18°C plus booster-by-immersion at 14°C 3-months later. **Blue stars**, infection-by-immersion at 14°C after booster of the VHSVS fish (VHSVS+). **B)** Levels of neutralizing Abs (NAbs) in plasma from: vaccination plus booster VHSVS (6 months after the first infection-by-injection); infection after booster VHSVS+ (VHSVS 2-days after a third infection); chronically infected with bacteria (bacteria); non-infected (NI) zebrafish and no added plasma (no plasma). The percentage of infected cells was calculated by the formula, 100 x number of cells with fluorescences above the threshold / total number of cells gated per well. *VHSV*-infected cell controls in the absence of added zebrafish plasma showed that 55% of the EPC cells were infected (fluorescent). The results were then expressed in percentage of neutralization by the formula, 100–100 x % of infected cells / 55. Each open circle corresponds to an individual zebrafish. **Red horizontal lines**, mean neutralization percentage values. **Dash line**, mean + 2 standard deviations of neutralization percentage of non-infected plasma (n = 8). *, mean percentage values significantly higher than non-infected mean at the p<0.05 level (Student t-test).

Three phenotypes were generated, namely primary infected zebrafish VHSV+ (naïve zebrafish 2-days after *VHSV* infection-by-immersion at 14°C), survivors of vaccination plus booster VHSVS (zebrafish surviving 3-months after a first *VHSV* infection-by-injection or vaccination at 18°C and a booster infection-by-immersion at 14°C 3-months later), and infected after booster VHSVS+ (VHSVS zebrafish 2-days after a third *VHSV* infection-by-immersion at 14°C) (S1 Fig). VHSVS showed between 70 to 90% of survivors in 3 experiments (Fig 1A, solid symbols). In contrast, only between 0 and 10% of VHSV+ survived (Fig 1A, open symbols). These results confirmed that vaccination at 18°C generate large numbers of the VHSVS phenotype [21, 22, 24, 31]. Six-months after vaccination (3-months after booster), 100% of VHSVS survived re-infection-by-immersion (third infection) at 14°C (VHSVS+) (Fig 1A, blue star symbols). RTqPCR performed 2-days after the third infection showed that VHSVS+ produced 740 ± 528-fold fewer N_*VHSV*_ transcript molecules than VHSV+ (n = 4 replicas per phenotype). The lower numbers of N_*VHSV*_ transcripts suggests that the defenses memorized in VHSVS inhibited the early replication of *VHSV* and explained subsequent 100% survival of this phenotype. In addition, all these data indicate that VHSVS had more memory defenses than those fish that were only vaccinated.

### Evaluation of specific adaptive memory responses

To study the presence of anti-viral specific antibody (Ab) adaptive responses (neutralizing Abs, NAbs) we performed *VHSV* micro-neutralization assays of the plasma of the same zebrafish used for the microarray experiments. As controls, plasma from both naïve fish (non-infected, NI) and survivors of bacterial infections were included. We observed that 8 of 10 VHSVS fish (80%) had NAbs levels significatively higher than those in NI fish (Fig 1B), thus confirming previous data [24] and suggesting these NAb levels could explain the survival rates of 70–90% (Fig 1A, solid symbols). VHSVS+ (*VHSV* infection after booster) did not significantly changed the percentage of fish with NAbs (Fig 1B), suggesting that this phenotype had sufficient defenses to respond early to the *VHSV* re-infection but not explaining its 100% survival. The specificity of the NAb responses was confirmed by the absence of NAb titers in plasma from survivors of bacterial-infection and NI fish (Fig 1B). In conclusion, because VHSVS+ fish showed a survival rate of 100% (Fig 1A, blue stars), despite only 54.5% of them having NAbs, we propose that other memory mechanisms contribute to their survival, in addition to NAbs. This data, prompted us to further explore the immunological status of the VHSVS phenotype using microarrays.

### Identification of modulated MultiPath Genes (mMPG)

To begin the transcriptomic analysis from the microarray data of VHSV+, VHSVS, and VHSVS+, we extracted those genes present in multiple pathways (MultiPath Genes, MPG) whose transcript expressions were modulated (up or down regulated). To perform the analysis, modulated MPG (mMPG) were arbitrarily defined as those that, **i)** were common to >6 pathways; **ii)** had >2 or <0.5 folds (thresholds) when comparing *VHSV*-infected phenotypes versus NI fish in at least one phenotype; and **iii)** were significantly different from one of the thresholds.

**Fig 2:**
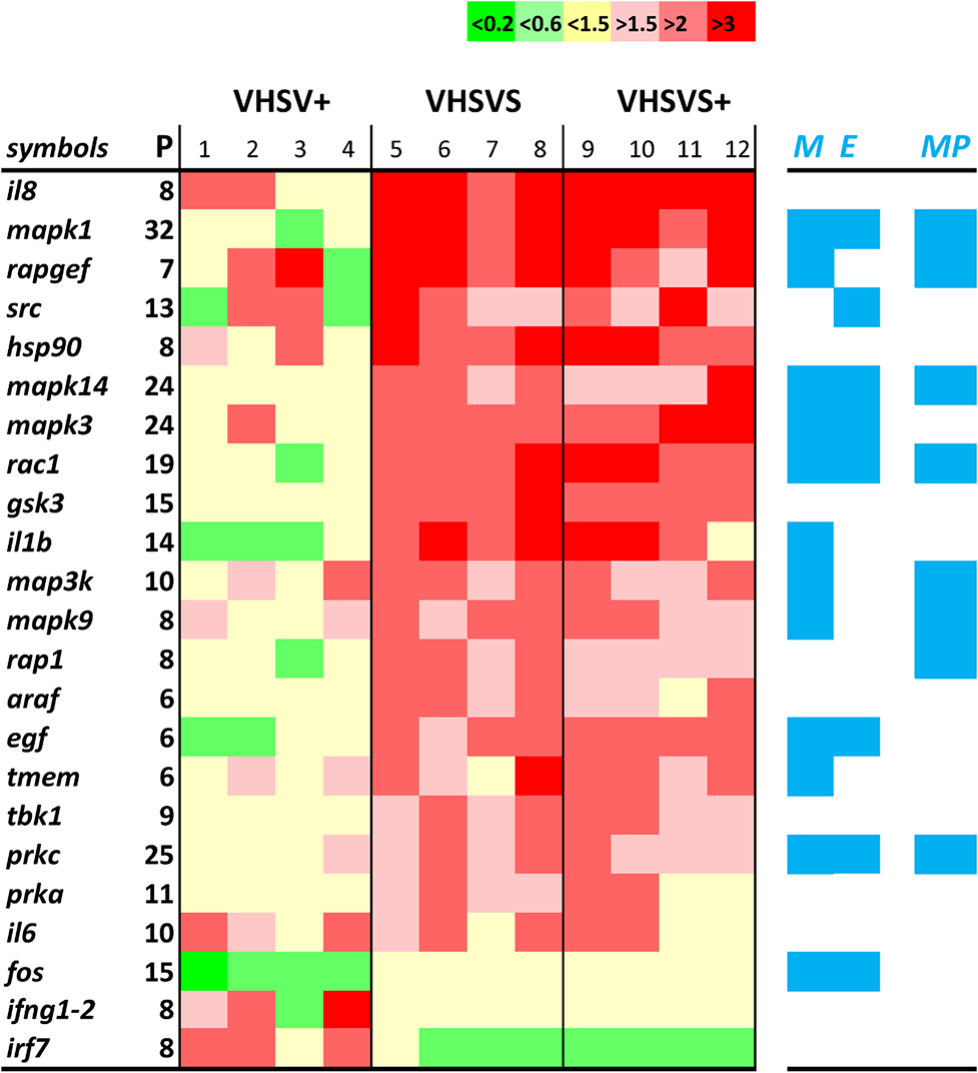
Heat map of Modulated MultiPath Genes (mMPG). The mMPG with folds >2 or <0.5 in at least one of the phenotypes, were ordered by the expression levels in VHSVS. Relative differential expressions were calculated versus NI fish. **Bright gree**n, <0.2. **Light green**, <0.66 and >0.2. **Yellow**, folds <1.5 and >0.66. **Light red**, >1.5 and <2. **Red**, >2 and <3. **Intense red**, folds >3. **P,** number of pathways in which the mMPG were present. **1–12**, biological replicates. **Blue M,** mMPG genes present in the “Mitogen activated protein kinase pathway” (MAPK). **Blue E**, mMPG genes present in the "EGFR1 signaling pathway". **Blue MP,** mMPG genes present in the novel 12MAPKS+5PIRP GS defined after Leading Edge Analysis (see S5 Table, red).

We detected 154 genes common to >6 in the 62 immune-related human/zebrafish orthologous pathways of the in-house microarray (S1 Table). Only 14.9% of these were mMPG in at least one of the phenotypes. Fig 2 shows that the mMPG were: **i)** the same genes for VHSVS/VHSVS+ and different for VHSV+, **ii)** generally upregulated in VHSVS/VHSVS+ but downregulated in VHSV+; and **iii)** related to innate immune signaling. Strikingly, mMPG included 5 mitogen-activated protein kinases (*mapk1*, *mapk14*, *mapk3*, *map3k*, *mapk9*) and 7 additional genes of the "MAPK signaling pathway" (*rapgef*, *rac1*, *il1b*, *egf*, *tmem*, *prkc*, *fos*). Most of these genes were upregulated in VHSVS/VHSVS+, while only *rapgef/mapk9/fos* were modulated in VHSV+, thereby supporting the relevance of the MAPK pathway for the VHSVS/VHSVS+ phenotypes. In addition, some of these mMPG (*mapk1*, *mapk14*, *mapk3*, *rac1*, *egf*, *prkc*, *fos*) were common to the "EGFR1 signaling pathway" and/or to the "epidermal growth factor receptor signaling network" [40], both involved in one of the most important pathways that regulates growth, survival, proliferation, and differentiation in vertebrate cells. Furthermore, pro-inflammatory interleukins, such as *il8/il1b/il6*, were upregulated in VHSVS/VHSVS+. The increase in the expression of these interleukins is among the hallmarks of the earliest innate immune system responses of fish to viral infections and was also reported in 1-month *SVCV* (spring viremia carp virus)-survivor zebrafish [25]. In addition, of special interest was the coordinated upregulation of *il1b* (pro-inflammatory cytokine) and *rac1* (transcription factor) since they are markers of cell populations with enhanced migration capacity [41].

The following pathways were most enriched in mMPG in all three phenotypes: "MAPK signaling" (12 mMPG), "T cell receptor signaling" (10 mMPG), and "interleukin 3" (10 mMPG). In addition, the "Toll-like receptor signaling", "interleukin 5" (a growth factor for B-cells), "interleukin 6" (a pro-inflammatory cytokine activated during the acute phase response and therefore related to *crp*), "hepatitis", "RIG-I-like receptor signaling", "B-cell receptor signaling" and "EGFR1 signaling" pathways contained 8–9 mMPG. The remaining pathways showed < 7 mMPG (not shown). Previous observations on the mMPG importance of MAPK signaling, and Toll-like, B-cell, T-cell and RIG receptors have also been described in 1-month SVCV-survivor zebrafish [25].

### Results of the Gene Set Enrichment Analysis

As the analysis of mMPG is dependent on the pathways selected and limited to those pathways with MPG, some complementary analysis was required. For instance, the pathways corresponding to "Complement and coagulation cascades", "Proteasome degradation", "Snare interactions in vesicular transport" and "Protein export" cannot be analyzed by the MPG method because they have no MPG. In addition, any analysis restricted to gene-by-gene expression may overlook biological effects arising from smaller but coordinated changes in interconnected genes. Furthermore, a statistical evaluation of the relative importance of the pathways or gene sets (GSs) was also needed. Therefore, we next focused on Gene Set Enrichment Analysis (GSEA) because it fulfilled all the above requirements. Thus, GSEA does the following: **i)** assigns an overall enrichment score (ES) to each GS or pathway; **ii)** normalizes ES (NES) by correcting for the number of genes in order to compare GSs, and **iii)** associates each GS with estimations of statistical significance.

A first GSEA was performed using its 10295 human GS data base and applying a strict False Discovery Rate (FDR) of <0.05 for significance. Because human and zebrafish gene symbols do not always coincide, only 2594 human GSs could be analyzed by this method (summarized results in S3 Table). In VHSV+, all the enriched GSs detected were downregulated, confirming the immunosuppression in lymphoid organs when zebrafish is infected with rhabdoviruses [22, 25] and the mMPG data (Fig 2). These GSs included many related to proteasome/antigen presentation (S3 Table, VHSV+ in red), and cell proliferation (S3 Table, VHSV+ in italics), thereby suggesting that these pathways are among the most important to block in order to favor initial viral replication. In sharp contrast, GSs-related to proteasome/antigen presentation (S3 Table VHSVS in red), and cell proliferation (S3 Table VHSVS in italics) were upregulated in VHSVS. Other GSs related to interferons (S3 Table, VHSVS in blue) and complement were downregulated in VHSVS, while few modulations were detected in VHSVS+ (S3 Table, VHSVS+). Therefore, these results confirmed the implication of proteasome/antigen presentation and cell proliferation detected by the mMPG analysis on the VHSVS phenotype.

A second analysis using the human/zebrafish orthologous pathway GSs and the zebrafish keyword-selected GSs from the in-house microarray (S1 Table) confirmed that "Proteasome degradation" (including the *psm* gene family) was upregulated in VHSVS/VHSVS+, while *crp*, *mx*, *nitr*, *ifn*, *mhc*, complement/"complement and coagulation cascades", and "Type II interferon signaling" were downregulated (Table 1, gene compositions in S4 Table). Of note, most of the GSs that were enriched belonged to the zebrafish keyword-selected GSs (*nitr*, *ifn*, *mhc*, *complement*, including those GS with some genes added such as *crp*, *mx*) rather than to the human/zebrafish orthologous pathway GSs ("Proteasome degradation", "complement and coagulation cascades", and "Type II interferon signaling"). On the other hand, MAPK- and EGFR-pathways were not enriched despite the upregulation of many of their mMPG (Fig 2). The results also confirmed that in VHSV+ many pathways were downregulated as found when using human GSs and as reported previously for *VHSV* [22] and *SVCV* [25] infections. Early immunosuppression could be induced by rhabdoviruses to favor their initial replication, a phenomena that has been described for other viruses [42–48]. In this respect, the non-virion NV protein of *VHSV* was recently identified as an extensive immunosuppressor viral protein in trout [49] and similar preliminary results were confirmed in zebrafish (unpublished). Alternatively, cell migration (as suggested by *il1b/rac1* upregulation) to the entry sites of *VHSV* (fins, skin, blood, etc), may cause a cell depletion in lymphoid organs, thus explaining the transcript downregulation in lymphoid tissues.

**Table 1:** Comparison of Normalized Enrichment Scores (NES) from GSEA of human/zebrafish orthologous GSs (gene symbols described in GEO's GPL17670).

Gene Sets (GSs):	NES:		
gene composition in S4 Table	VHSV	VHSVS	VHSVS+
**proteasome degradation (*psm*)**	**-3.04	**1.77	1.49
**nitr, novel immune-type receptors**	*1.50	*-1.32	-1.01
mhc, membrane histocompatibility complex	-1.04	**-1.69	**-1.59
complement and coagulation cascades	0.91	**-1.76	**-2.12
type II interferon signaling (ifng)	-1.19	**-1.80	**-1.65
com, complement-related proteins	0.79	**-2.12	**-2.35
**mx, myxovirus-induced proteins**	*1.50	**-2.15	**-2.28
ifn, interferons	1.11	**-2.20	**-1.92
**crp, c-reactive proteins**	-0.81	**-2.89	**-3.14

The list of unique genes with their corresponding fluorescence values from pooled lymphoid organs from 3 zebrafish per replica per phenotype (n = 4 replicas), was used for GSEA comparisons. The GSEA software was then applied to the Gene Sets (GSs) defined in the in-house microarray (gene symbols described in GEO's GPL17670, see S1 Table for a GS summary). GS Enrichment Scores (ES) were normalized for their number of genes (NES) and their significance was assessed by using 1000 gene permutations to estimate null distributions. The data were ordered from the highest to lowest NES of VHSVS. The differential expressions for the 3 phenotypes were calculated versus NI zebrafish. The remaining GSs did not show significant NES.

**+**, NES correlating with the first phenotype in the comparison.

**-**, NES correlating with NI in the comparison.

** (bold numbers), FDR q value < 0.05.

*, FDR q value <0.25.

**bold**, GSs containing multigene families.

**Fig 3:**
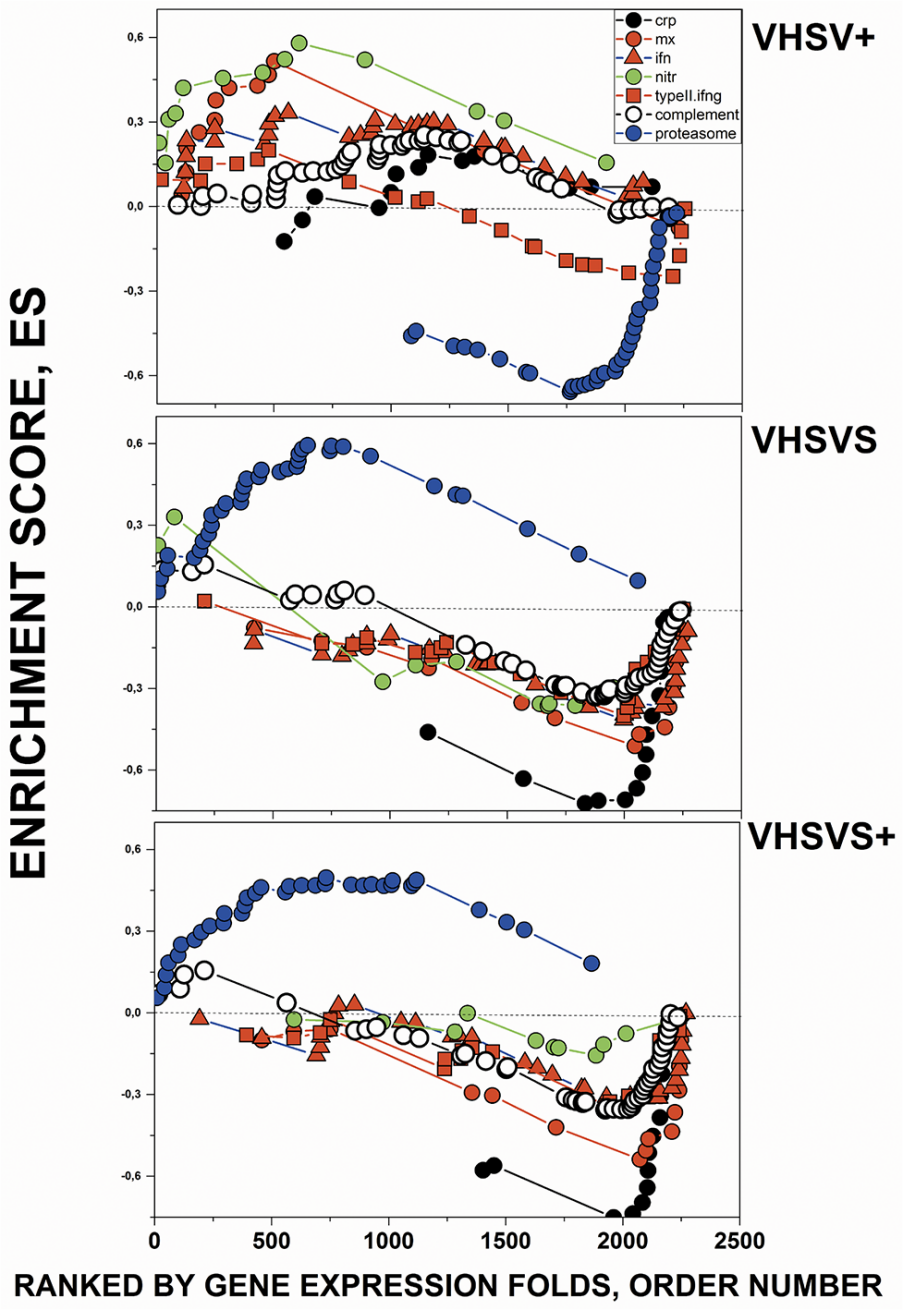
Comparison of Enrichment Scores (ES) for individual genes from GSs with significant NES. Some of the most enriched human/zebrafish orthologous GSs of Table 1 were compared by their corresponding individual gene enrichment plots (ES per gene in the Y axis *versus* ranked list of genes ordered from their highest to lowest differential expression folds in the X axis). Those genes ranked first in the X axis correlated with the first phenotype of the comparison (VHSV+, VHSVS, VHSVS+) while those at the end correlated with NI. **Black circles**, *crp* (c-reactive protein) keyword-selected GSs with added complement components (genes listed in S4 Table). **Red circles**, *mx* (myxovirus-induced protein) keyword-selected GSs with added interferon genes (S4 Table). **Green circles**, *nitr* (novel immune-type receptor) keyword-selected GSs (S4 Table). **Blue circles**, “Proteasome degradation” WIKI pathway (S4 Table). **Open circles**, “complement and coagulation cascades” KEGG pathway (S4 Table). **Red squares**, “Type II interferon signaling (*ifng*)” WIKI pathway (S4 Table). **Red triangles**, *ifn* (interferon) keyword-selected GSs (S4 Table). The complete list of zebrafish GS genes in the in-house microarray and their corresponding probe sequences can be found at GEO’s GPL17670.

The plots of the most enriched GSs in Table 1 (Fig 3), graphically illustrated the following: **i)** the similar individual gene ES profiles between VHSVS/VHSVS+, including upregulation of genes in the "Proteasome degradation" pathway and downregulation of those in *crp*, *mx*, *nitr*, *ifn*, Complement and "Complement and coagulation cascades"; **ii)** the opposite behaviors between VHSVS/VHSVS+ and VHSV+ ES; and **iii)** the small changes in the genes belonging to "Type II interferon signaling" GS in the 3 phenotypes, thus suggesting a marginal role for *ifng* during zebrafish infection and survival.

We then applied 2 alternative approaches to further study the results obtained. On the one hand, because some of the most enriched GSs contained multigene families (those named *crp*, *mx*, *nitr*, *psm*) (Table 1 in red), we analyzed the differential expression of the multigene components to explore possible relations with trained immunity. On the other hand, we addressed why many mMPG were upregulated in VHSVS (Fig 2), while most of the corresponding human/zebrafish orthologous pathways were unchanged or downregulated (Table 1 and Fig 3).

### Comparative differential expression of individual genes of *crp*, *mx*, *nitr* and *psm* multigene families

**Fig 4:**
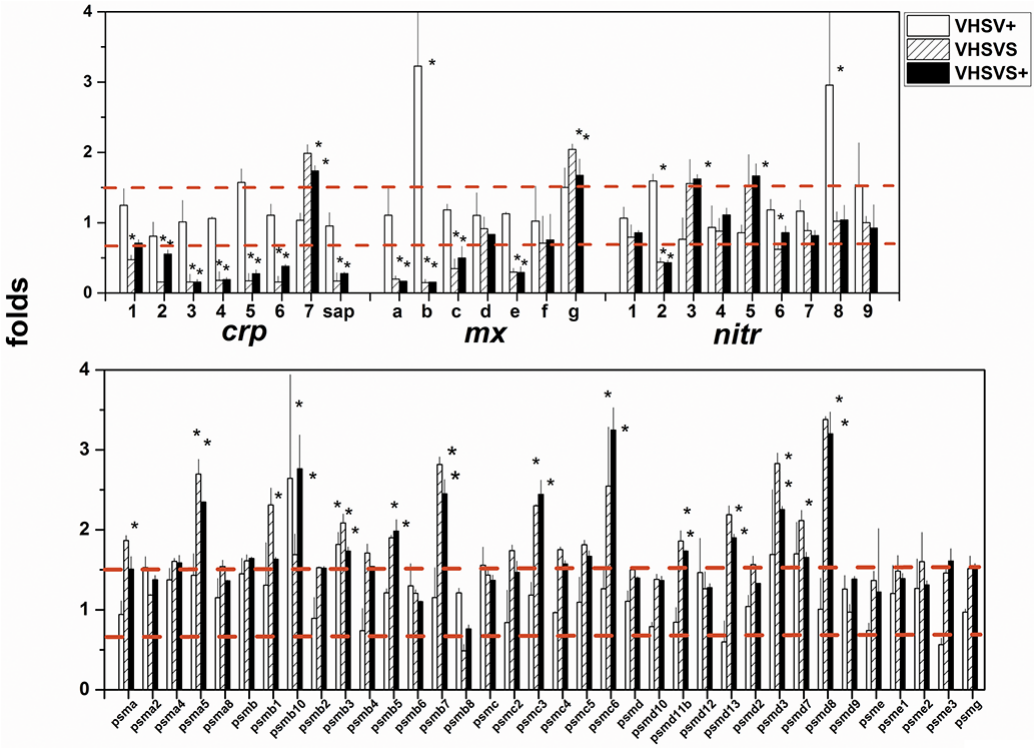
Differential expression profiles of individual *crp*, *mx*, *nitr* and *psm* genes from multigene family GSs. After normalization and reduction to a list of unique genes, folds were calculated for each gene using the formula: fluorescence of each *VHSV*-infected replicate / mean fluorescences from the NI replicates (n = 4). Means and standard deviations were then obtained for each gene and outliers were removed to obtain final folds (n = 4). **A,** *crp*, *mx* and *nitr* multigene families. **B**, *psm* multigene family. **Open bars**, VHSV+. **Hatched bars**, VHSVS. **Black bars**, VHSVS+. **Red horizontal bars**, 1.5- and 0.66-fold thresholds. *, significantly >1.5 or <0.66 at the p = 0.05 level (Student t-test).

C-reactive protein (CRP) and serum amyloid P-component form a family of acute phase pentraxin genes (*crp*, *sap*, respectively) which are involved in the rapid secretion of soluble proteins after bacterial infection/injury in most animal species, including fish [50]. Pentraxins are characterized by their capacity to bind to a wide range of phospholipid molecular heads in a Ca^++^-dependent manner [51, 52]. Phospholipid bound CRP activates the classical complement pathway by binding to C1q [53]. The genome of zebrafish codes for 8 *crp*-related genes (*crp1-7* and *sap*) which show differential expression throughout tissues [54]. Using comparisons with NI zebrafish and arbitrarily fixing folds at 1.5/0.66 thresholds for significance, we found that *crp* 1–6 and *sap* (all except *crp7*), are downregulated in VHSVS/VHSVS+ while they remain unmodulated in VHSV+ (Fig 4). These results were validated by RTqPCR with a Pearson’s coefficient of 0.85 (CRP in S3 Fig). This is the first description of typical bacteria-dependent *crp* responses in multi-*crps* in viral-infected VHSVS/VHSVS+ phenotypes. To date, virally-induced *crp* upregulation had been only reported in trout after oral vaccination against infectious pancreatic necrosis virus [55].

The MX proteins are coded by members of a family of *ifn*-inducible genes (*mx*) shortly after viral infection [56, 57]. Mammals have 2–3 *mx* genes, while trout have 3 [58] and zebrafish 7 (*mxa-g*) [59]. Virus inhibition by *mx* has recently been demonstrated in fish [60–62], despite unsuccessful earlier attempts [63]. Furthermore, *mx* upregulation has been reported in DNA-vaccinated fish [64, 65], including zebrafish [21, 22] but studies on the expression of multiple zebrafish *mx* isoforms have not been reported yet. We show here that *mxa*,*b*,*c*,*e* were downregulated while *mxg* was upregulated in VHSVS/VHSVS+ (Fig 4). Only *mxb* was upregulated in VHSV+. These results were validated by RTqPCR with a Pearson’s coefficient of 0.61 (MX in S3 Fig).

Novel immune-type receptor (*nitr*) genes belong to multigene families encoding transmembrane proteins containing immunoglobulin-like variable domains with a high degree of sequence variation [66, 67]. A maximum haplotype of 36 *nitr* zebrafish genes can be grouped into 12 families, including inhibitory (*nitr1-8*, having immune receptor tyrosine inhibition motifs ITIM) and activating (*nitr5*, *nitr7a*, *nitr9*, *nitr10*, *nitr11a*, *nitr12*) receptors [68]. A high level of individual *nitr* heterozygosity is reflected in haplotype variations, allelic polymorphisms, and isoforms [68]. Several *nitr* families expressed in teleost NK cell lines with alloreactive specificity have been related to trained immunity [69, 70]. *Nitr3*,*5* were upregulated in VHSVS/VHSVS+ while only *nitr2*,*8* were upregulated in VHSV+ (Fig 4). Although there is no explanation of the functions of these *nitr*, their complexity, immunoglobulin-like structures, and individual polymorphisms together with preliminary evidence are consistent with their participation in trained immunity.

Proteasomes/immuno-proteasomes are formed by multi-protease subunit complexes that degrade proteins inside cells [71, 72], perhaps with tissue-specificity [73]. Proteasomes contain a central proteolytic core barrel made up of constitutive PSM-related proteins (i.e.: PSMB5,6,7) coded by their corresponding genes (*psma*,*b*,*c*,*d*,*e*,*f*,*g*). Upon interferon induction additional PSM proteins (i.e. PSM8,9,10) are incorporated to immunoproteasomes to substitute the otherwise constitutive subunits, thus leading to the acquisition of novel proteolytic activities. In zebrafish, both human orthologous and unique *psm* genes have been described [74]; however, their distinct functionalities are unknown. Many *psm* genes were upregulated in VHSVS/VHSVS+ (i.e.: *psma1*,*5*, *psmb1*,*5*,*7*, *psmc3*,*6*, *psmd3*,*7*,*8*,*11b*,*13*) (Fig 4) but none were modulated in VHSV+. These results confirm that the implication of the "Proteasome degradation" pathway in VHSVS/VHSV+ is caused by *psm* genes and they highlight the relevance of these genes for maintaining resistance to re-infection.

All multigene families were similarly modulated in VHSVS/VHSVS+ (Table 1 and Figs 3 and 4), thereby strongly suggesting that they are involved in the maintenance of these phenotypes. Furthermore, these gene families have common characteristics making them candidates as trained immunity molecules or candidates contributing to resistance to re-infection. In this regard, each multigene family contained closely related genes with variation in sequences and had some genes that were constantly expressed (up or downregulated) correlating with possible VHSVS/VHSVS+ memories. However, their precise function remains to be elucidated. Thus, these findings pave the way for future studies focused on individual gene sequence polymorphisms, variations in their tissue distribution, molecular binding specificity of the different isoforms (i.e. CRP/phospholipids), and/or mechanisms that generate different responses, among others.

The innate defenses that VHSVS accumulated are so strong that 2-days after re-infection (VHSVS+) only minor transcriptional changes were detected despite 100% survival. The VHSVS phenotype does not need additional proteins to maintain that viral barrier. The downregulation of genes in VHSVS might occur because they are part of feedback mechanisms to prevent excessive host cell damage, once the corresponding proteins have reached protective levels in the tissues. The prior accumulation of the corresponding multi-protein molecules might be part of such a defensive strategy. On the other hand, although some of the downregulated genes might be inhibitors, like *nitr2*,*6* [68] (in which case downregulation will increase anti-viral functions), no such inhibitory properties have been described in the *crp*, *mx* or *psm* multigene families. Nevertheless, the abundance of downregulated genes and pathways in the VHSVS/VHSVS+ phenotypes remains intriguing.

### Leading Edge analysis of enriched GSs

**Fig 5:**
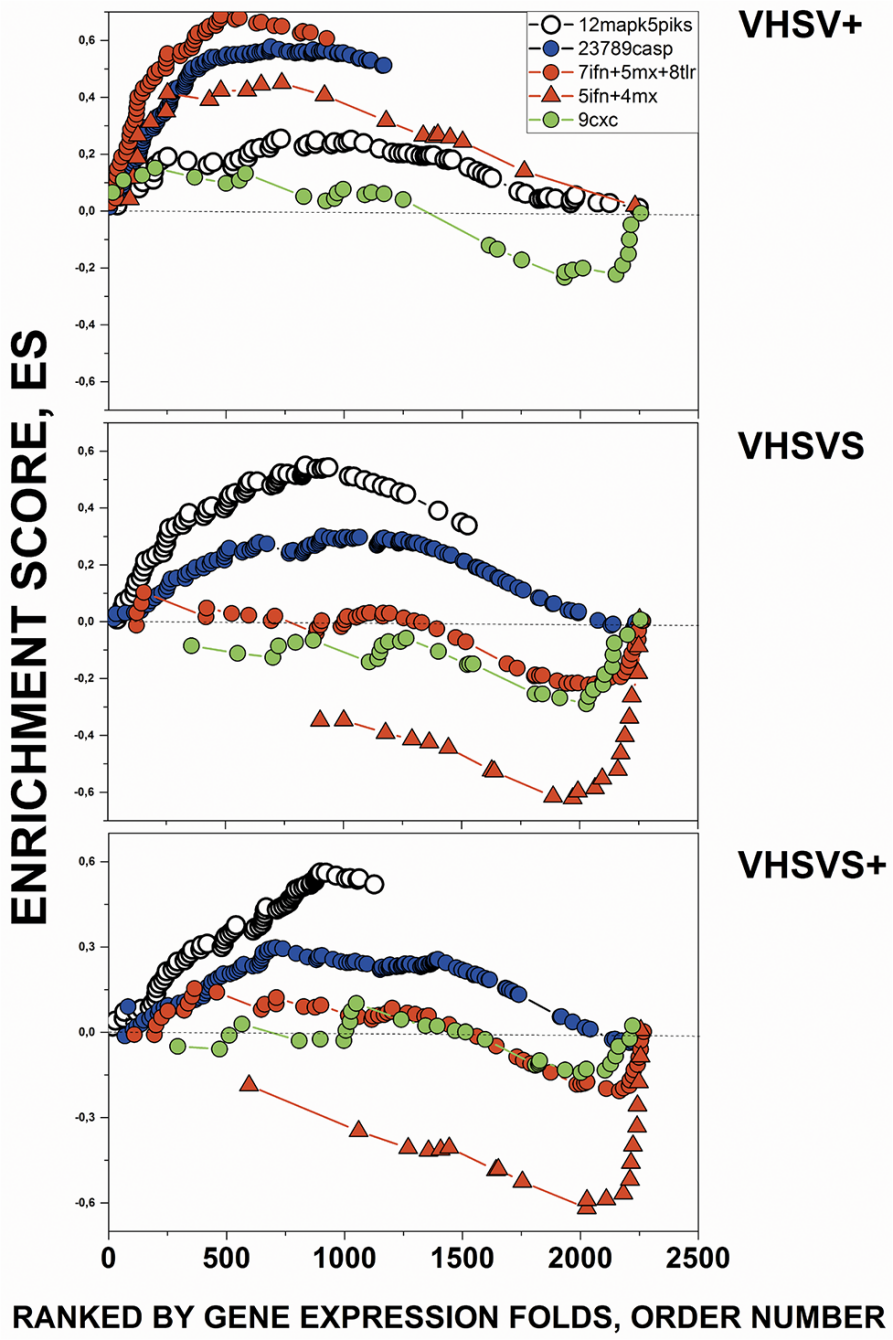
Comparison of gene ES from the Leading Edge novel GSs with significant NES. The novel GSs with significant NES are listed in Table 2 (gene compositions in S5 Table). **Open circles**, novel GSs containing 12 *mapks* and 5 *pirp* (phosphoinositide-related proteins). **Red circles**, novel GSs containing 8 *tlr*, 5 *ifn* and 5 *mx* genes. **Red triangles**, novel GSs containing 5 *ifn* and 4 *mx*. **Blue circles**, novel GS containing 5 *caspases*. **Green circles**, novel GS containing 9 chemokines (*9cxc*). The complete list of genes for each of the novel 14 GSs are described in the S5 Table.

As the results of the first GSEA were based on human GSs and the second on human/zebrafish orthologous GSs and although many of the enriched GSs were derived from zebrafish mRNA (*nitr*, *mhc*, *complement*, *mx*, *ifn*, *crp*), there may be more unique zebrafish GSs that were not analyzed. To identify novel candidates for zebrafish GSs, the results of the second GSEA were used for a Leading Edge analysis. Our results indicated 14 novel gene clusters of possible interconnected zebrafish genes (see S5 Table for gene composition). Therefore, we performed a third GSEA using these gene clusters as GSs. Novel potentially co-upregulated genes characteristic of VHSVS/VHSVS+ (here called 12MAPKS+4PIRP, containing *mapks* and phosphoinositide receptor protein-related genes) and of VHSV+ (8TLR+7IFN+5MX, containing *tlr*, *ifn* and *mx* genes and 23789CASPS containing many caspases) were thus identified (Table 2). The plots corresponding to the enriched novel GSs (Fig 5) confirmed the similar GS profiles between VHSVS/ VHSVS+, and the contrast between VHSVS/VHSVS+ and VHSV+. Furthermore, they also showed an small increase in 12MAPKS+4PIRP of VHSVS+ relative to VHSVS (Fig 5, open circles), a pattern that mimics a typical behavior of trained immunity. In addition, they revealed an example of GS without significant alterations among phenotypes (Fig 5, 9CXC, green circles).

**Table 2:** Comparison of NES from GSEA of novel GSs derived from Leading Edge Gene Analysis (gene symbols described in S5 Table) of the data summarized in Table 1

Novel GSs	NES:VHSV+	VHSVS	VHSVS+
**12MAPKS+5PIRP**	1.04	**1.81	**1.83
**23789CASPS**	**2.55	1.02	1.00
**8TLR+7IFN+5MX**	**2.72	-1.17	*-1.35
**5IFN+4MX**	*1.44	**-2.84	**-2.85

The results show the significant NES among the 14 novel GSs proposed by the Leading Edge Gene Analysis. The differential expressions were calculated versus NI zebrafish. The numbers before the gene names indicate the total number of these genes in each novel GS. The novel GS names indicate some of the majoritary genes which form part of the novel GSs (The S5 Table shows the corresponding gene symbols of all novel GSs).

**Bold Novel GSs**, novel GSs proposed by Leading Edge analysis of GSEA summarized in Table 1. **MAPKS**, mitogen-activated protein kinases.

**PIRP**, phosphatidyl-inositol related gene proteins.

**CASPS**, caspases.

**TLR**, Toll-like receptors.

**IFN**, interferons.

**MX**, myxovirus-induced proteins.

**+**, NES correlating with the first phenotype in the comparison.

**-**, NES correlating with NI in the comparison.

** (bold), FDR q value < 0.05.

*, FDR q value < 0.25.

The 12MAPKS+4PIRP novel GS (Table 2, Fig 5) had 8–43% common genes with human/zebrafish orthologous MAPK-related pathways, 30.7% with "T cell receptor signaling", 27.7% with "B-cell receptor signaling", 24.5% with mMPG (Fig 2), 40% with "Integrin-mediated cell adhesion" and 25% with "EGFR1 signaling" pathways. The 8TLR+7IFN+5MX novel GS contained 58.8% genes shared with *mx*. The 23789CASPS novel GS shared 31–50% genes with apoptosis-related pathways, and 40.3% with the "Interleukin 6" and 24% with "EGFR1 signaling" pathways. Therefore, these results showed that the novel GSs contained new combinations of related genes from various human/zebrafish orthologous pathways, and explained some of the previous observations using human/zebrafish orthologous pathways, such as the low number of upregulated pathways in VHSVS/VHSVS+ (Table 1), despite mMPG upregulation (Fig 1). Nevertheless, the 12MAPKS+4PIRP novel GS together with the "proteasome degradation" pathway (Table 1) were the only GSs that remained upregulated in VHSVS/VHSVS+. Similarly, VHSV+ was described not only by mMPG and pathway downregulations but also by upregulated 8TLR+7IFN+5MX (Toll-related) and 23789CASPS (apoptosis-related) novel GSs. These results suggest that interpreting zebrafish transcriptional results only by using human or human/zebrafish orthologous pathway GSs might not be accurate. Novel pathway GSs might provide a better explanation of the coordinated behavior of some genes during resistance to infections in these primitive vertebrate models. Further fine-tuning of the relationships among the genes of these proposed novel pathways for zebrafish will be required to confirm their physiological significance.

### Transcript profiles and flow cytometry analysis of immune zebrafish cells

Given the unavailability of anti-protein reagents to detect markers of immune zebrafish cells, we listed some of the genes related to Th1, Th2, Th17 (T-helper), Treg (T-regulatory), B, BZ (B-cells expressing IgM or IgZ, respectively), dendritic, CTL (cytotoxic), NK (natural killer), macrophages, and neutrophil cells (genes described in S6 Table), with the purpose to be used as cell GSs for GSEA.

Neutrophil/macrophages were the only cell types whose ES were positive in the 3 phenotypes, albeit with FDR > 25% (Table 3). These results correlate with the known involvement of neutrophil/macrophages in many fish pathogen infections [75–78] and with the mMPG upregulation in the *il3* pathway, which is crucial for neutrophil/macrophage differentiation. On the basis of these results and given that most other cell types were reduced in lymphoid organs in VHSVS or VHSVS+ compared to VHSV+ we postulate that neutrophil/macrophages are responsible for most of the positive transcriptional profiles in these 2 phenotypes. CTL decreased in all the phenotypes, more in VHSVS/VHSVS+ than in VHSV+ while Th2, B, and NK cells decreased only in VHSVS (Table 3). CTL depletion might reflect either downregulation of transcripts or cell migration from the lymphoid organs to the *VHSV* entry sites; however the lack of anti-protein reagents against the markers of these transcripts precludes validation of these results.

**Table 3:** Comparison of NES from GSEA of GS defining distinct immune cell types (gene symbols described in S6 Table)

Cell type GS	n° genes per GS	NES:VHSV+	VHSVS	VHSVS+
NEUTROPHIL	16	1.27	0.61	0.81
MACROPHAGES	31	1.09	0.29	0.36
TH17	37	0.88	-0.8	0.54
DENDRITIC	10	0.64	-0.98	-1.1
BZ	23	0.62	-1.08	0.4
TH1	30	0.89	-1.18	0.4
TH2	31	0.93	-1.22	0.37
B	23	0.58	*-1.35	-0.84
NK CELLS	35	1.15	*-1.4	-0.84
TREG	25	0.88	*-1.54	-0.99
CTL	12	*-1.16	**-2.14	**-1.68

To estimate the different immune cell activities, new Gene Sets (GSs) were defined (gene compositions described in S6 Table). To define genes for each cellular type, activating, membrane and secreted genes were selected and added to the GS from data obtained from various sources. The resulting GSs shown by their symbols in the S6 Table, were used as inputs for GSEA analysis. The NES values of each cellular type ordered by those in VHSVS are shown. The differential expressions were calculated versus **NI**, non-infected zebrafish.

**Th1**, T helper 1 cells.

**Th2**, T helper 2 cells.

**Th17**, T helper 17 cells.

**Treg,** T regulatory cells.

**B cells,** IgM-producing cells.

**BZ** cells, IgZ-producing cells.

**Dendritic**, dendritic cells.

**Cytotoxic,** antigen-specific cytotoxic cells.

**NK cells**, natural killer cells.

**Macrophages**, monocyte and macrophages.

**Neutrophil,** neutrophil and granulocyte cells.

**+**, NES correlating with the first phenotype in the comparison.

**-**, NES correlating with NI in the comparison.

** (bold), FDR q value<0.05.

*, FDR q value <0.25.

**Fig 6:**
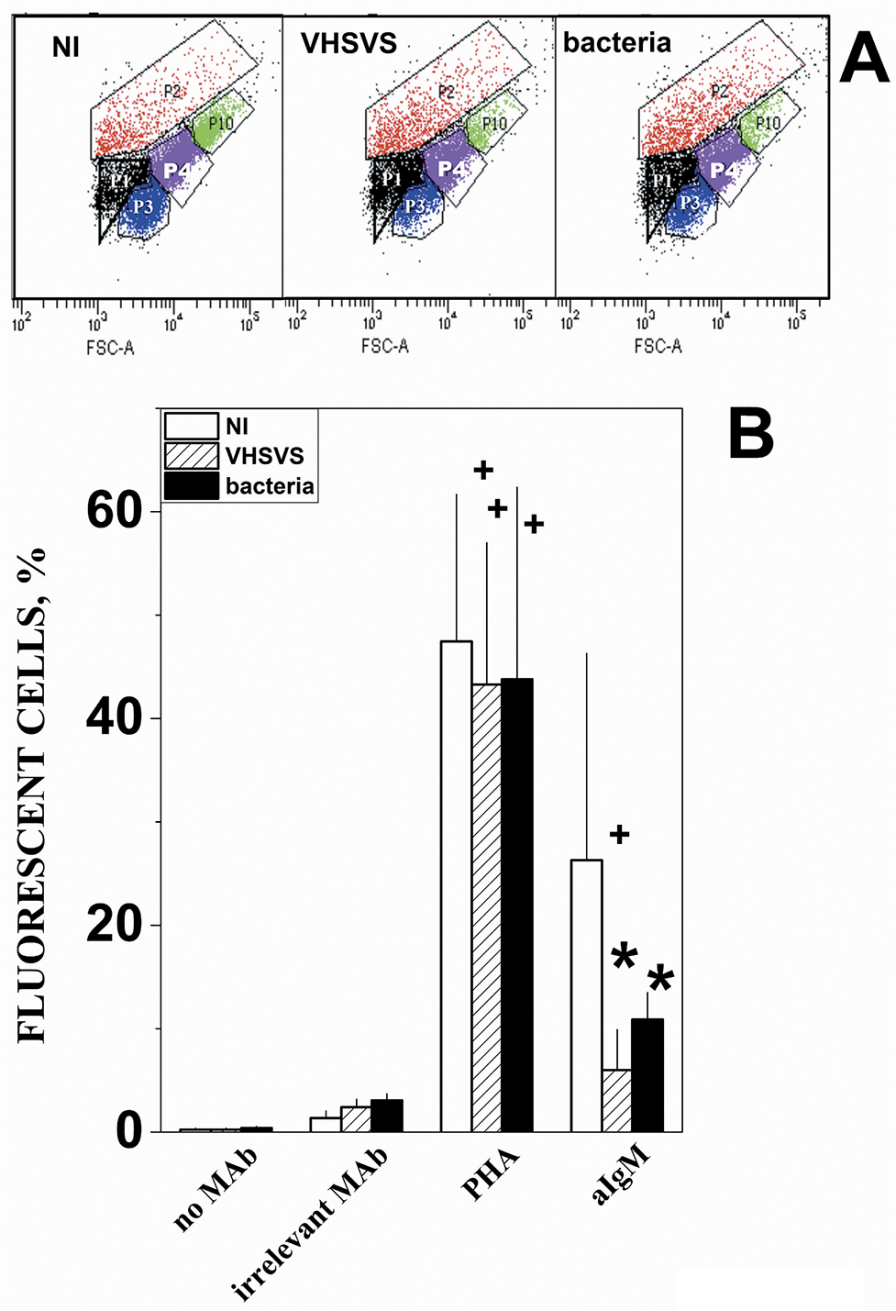
Flow cytometry scatter plots (A) and distribution of IgM+ cells in lymphoid organs (B). **A)** Representative FSC/SSC scatter plots used to define cellular populations and composition (mean ± standard deviations, n = 9) in pooled lymphoid organs from different phenotypes. **P1 black** (13.2 ± 5.5%) and **P3 blue** (12.6 ± 4.9%), damaged cells and/or cellular debris identified using sonicated cells. **P2 red** (20.4 ± 21.2%). **P4 purple** (40.8 ± 21.5%), lymphocytes as determined before [39]. **P10 green** (4.3 ± 2.7%). **B)** Cells from lymphoid organs were pooled from 3 NI (open bars), VHSVS (hatched bars) or bacterial-survivor (black bars) zebrafish phenotypes. Cells were stained with no MAb (in the absence of any MAb), an irrelevant MAb, FITC-phytohemaglutinin (PHA) and zebrafish crossreacting anti-trout IgM MAb 6B7 (aIgMt). The percentage of cells above the threshold fluorescence in the P4 population was calculated by the formula: 100 x number of cells in P4 with fluorescence above the threshold / number of P2+P4+P5 cells. Mean and standard deviations were represented (n = 2). **+**, significantly different from the staining with the irrelevant MAb. *, significantly different from irrelevant MAb or NI stained with anti-IgM.

In VHSVS/VHSVS+ mucosal *igz* was upregulated when compared to *igm* but this upregulation was not observed in VHSV+, thereby suggesting that the IgZ^+^ cells were more important for survival than the IgM^+^ ones (S4 Fig). To validate the proteins corresponding to the transcriptional profiles mentioned above, attempts using trout anti-IgM crossreacting with zebrafish IgM were made to estimate IgM^+^ cell count by flow cytometry. Results showed that in VHSVS and in bacterial-survivors, IgM^+^ cells in lymphoid organs were reduced to 5–10% compared to ~ 25% in NI fish, while the PHA^+^ cell population remained constant (~ 40% of the cell population) in the 3 phenotypes (Fig 6). These results confirm the downregulation detected in the B cell transcripts from VHSVS (Table 3). VHSVS B-cells may have secreted IgM to the blood to become IgM^-^, since the PHA^+^ population remained stable in the lymphoid organs. Alternatively, the results could also be explained by IgM+ cell migration to the blood or the skin and a corresponding increase in other PHA^+^ cell types in the lymphoid organs.

## Conclusions

The memory build up described here may explain the exceptional resistance of the VHSVS phenotype to *VHSV* re-infection. Such resistance could be attributable not only to the well known constitutive levels of blood NAbs but also to those corresponding to the proteins coded by several multigene families (*crp*, *mx*, *nitr*, *psm*). Constitutively modulated levels of multigene family memories may produce faster responses, widen pathogen recognition and generate synergies to form a formidable barrier against re-infection. Surprisingly, *VHSV* re-infection did not induce extensive additional transcriptional changes in VHSVS, most probably because the existing defenses inhibited early viral replication. Therefore, rather than having an increased Ab-binding efficiency or a faster specific secondary response, such as occurs in mammals, zebrafish may maintain long-term specific (NAbs) and non-specific barrier memories (*mapk*, *psm*, etc.) ready for a second pathogen encounter. Our results open up avenues for research into the new defensive functions against viral infections of these known multigene families. Questions to be addressed by such studies include individual gene polymorphisms, distribution among tissues, regulation of multigene expression and non-specific cross protection to heterologous pathogens. Among others, trained immunity in fish may result from some of these multigene functions since, for instance *nitr* genes have been related to long-term NK cells, a hallmark of mammalian trained immunity [79]. In conclusion, here we have revealed several adaptive characteristics of multigene families as unforeseen properties of innate survival mechanisms in zebrafish. Given their primitive immunological system among vertebrates (no IgG switch, no IgM maturation, mucosal IgT/IgZ, phagocytic B-cells, etc), fish species are suitable models in which to further study these phenomena. For instance, to show non-specific cross protection related to trained immunity, VHSVS should also be tested for resistance to challenge with unrelated fish viruses in future experimentation.Vaccine development is also expected to benefit from these lines of investigation.

## Supporting Information

S1 Fig Generation of zebrafish phenotypes by primary infection (VHSV+), vaccination plus booster (VHSVS) and infection after booster (VHSVS+). **VHSV+,** primary infected zebrafish were first acclimatized to 14°C (**yellow horizontal bars**) over 7 days before being immersed for 2 h in 10^7^ focus-forming units (ffu) of *VHSV* per ml (**yellow vertical arrow**). Two days later, lymphoid organs (head kidney and spleen) were harvested and pooled from 3 zebrafish per biological replica (**red vertical arrow**). **VHSVS,** vaccinated plus booster zebrafish were first intraperitoneally injected (vaccinated) with 10^6^ ffu of *VHSV* in 10 μl volume (**green vertical bar**) and maintained for 1 month at 18°C (**green horizontal bar**). The survivors were then maintained for 2 months at 24–26°C (**blue horizontal bars**), acclimatized to 14°C, challenged by immersion in *VHSV* at 14°C as in VHSV+ (**yellow horizontal and vertical bars),** and maintained for 1 month at 14°C to record mortality. The survivors were then maintained for 2 additional months at 24–26°C (**blue horizontal bars**). At this point, lymphoid organs were harvested and pooled from 3 zebrafish per biological replica (**red vertical arrow**). **VHSVS+**, infected after booster VHSVS fish were acclimatized to 14°C, infected-by-immersion in *VHSV* at 14°C as in VHSV+ (**yellow horizontal and vertical bars),** and lymphoid organs were harvested 2-days after infection (**red vertical arrow**) as described above. **Horizontal arrow**, aproximated time in months. Four biological replicates of 3 pooled zebrafish per replica were made for each phenotype.
(EPS)

S2 Fig VENN diagram between non-targeted commercially available microarray and the pathway/keyword sections of the in-house immune-targeted microarray used in these studies. The VENN diagram compared unique accession numbers between the non-targeted zebrafish ID19161 platform microarray of Agilent vs2 (43803 probes, 37464 unique accession numbers) and our in-house immune-targeted microarray platform ID47562 (14540 probes, 12391 unique accession numbers). The software from BioInfoRx (http://apps.bioinforx.com) was used to derive the VENN diagram. The circle surfaces are proportional to the number of unique probes. **Blue**, non-targeted microarray corresponding to Agilent's platform ID19161. **Red**, pathway and keyword sections of our in-house immune-targeted microarray corresponding to Agilent's platform ID47562.
(EPS)

S3 Fig Microarray hybridization and RTqPCR fold comparison of differentially expressed *crp* and *mx* family genes. Microarray folds of the differentially expressed CRP and MX multigene families from S4 Table were compared with the corresponding folds obtained by RTqPCR as described in Methods. To increase clarity, only the means (n = 3–4) were represented. **Black ○,** Mean folds from lymphoid organs from vaccination and booster VHSVS. **Red ○**, Mean folds from lymphoid organs from infection after booster VHSVS+.
(EPS)

S4 Fig Modulated IgM and IgZ gene transcripts. The relative differential expression was calculated with respect to NI. **Bright gree**n, <0.2. **Light green**, <0.66 and >0.2. **Yellow**, folds <1.5 and >0.66. **Light red**, >1.5 and <2. **Red**, >2 and <3. **Intense red**, folds >3. **1–12**, biological replicates.
(EPS)

S1 Table Gene Sets (GS) selected for the in-house microarray targeted to zebrafish immune-related genes (Agilent's ID 47562). *Red*, Top enriched GSs by GSEA of Table 1 (gene composition in S4 Table). Gene composition of all GSs in GEOs GPL17670.
(DOCX)

S2 Table List of primers used for RTqPCR. RTqPCR was used to validate some microarray results using selected differentially expressed genes and to evaluate *VHSV* replication levels by N_*VHSV*_ (see methods). Forward and reverse primers amplifying 100–120 bp were designed using the Array Designer 4.3 program (Premier Biosoft Palo Alto CA, USA). The *rplp0* gene was used as normalizer gene.
(DOCX)

S3 Table Significant Normalized Enrichment Scores (NES) obtained by using GSEA of human GSs from the GSEA database. The list of unique genes with their corresponding normalized mean fluorescent values from 4 biological replicas of pooled head kidney + spleens from 3 zebrafish per replica per phenotype, were used for GSEA. GSEA was performed using the 10295 human GS from its web (msigdb.v4.0.symbols.gmt). GS Enrichment Scores (ES) were normalized for their number of genes (NES) and their False Discovery Rates (FDR) significance assessed by using 1000 gene permutations to estimate null distributions. Only the data with FDR < 0.05 were tabulated and ordered from the highest to the lowest NES. Only 2594 human GS passed the human/zebrafish symbol filter and resulted in the identification of enriched GS. **+ positive**, NES that correlate with the first phenotype in the comparison.—**negative**, NES that correlate with NI in the comparison. The rest of GSs did not show significant NES. **red bold**, proteasome/antigen presentation-related GS. ***Italics***, GS related to cell proliferation. **Green bold**, Apoptosis regulation. **Blue bold**, interferon-related. **Black bold**, complement and coagulation cascades.
(DOCX)

S4 Table Gene composition of the top GSs from GSEA of Table 1. Due to the small number of genes in the GSs defined by the *crp* and *mx* keywords, other related genes were added to reach the gene number requirements for estimation of significance.
(DOCX)

S5 Table Gene composition of novel GSs proposed by clustering the Leading Edge enriched genes according to the GSEA results of Table 1. **Red**, significantly enriched novel GSs (Table 2).
(DOCX)

S6 Table Gene composition of the GSs defining immune cell markers. Membrane, activating and secreting genes, were selected to design cell GSs from different sources. The selected genes were then filtered by its presence on the in-house microarray and the resulting gene lists were used as input for GSEA. **Th1**, T helper 1 cells. **Th2**, T helper 2 cells. **Th17**, T helper 17 cells. **Treg,** T regulatory cells. **B,** IgM producing cells. **BZ,** IgZ producing cells. **Dendritic**, dendritic cells. **Cytotoxic,** antigen-specific cytotoxyc cells. **NK**, natural killer cells. **Macrophages**, monocyte and macrophages. **Neutrophil,** neutrophil and granulocyte cells.
(DOCX)
