# Supplementary material for: Innate Multigene Family Memories Are Implicated in the Viral-Survivor Zebrafish Phenotype
Source: PLoS One. 2015 Aug 13;10(8):e0135483. doi: 10.1371/journal.pone.0135483 (PMC4535885; doi:10.1371/journal.pone.0135483)
Supplement: S1 Table — Red, Top enriched GSs by GSEA of Table 1 (gene composition in S4 Table). Gene composition of all GSs in GEOs GPL17670. (DOCX) [file pone.0135483.s005.docx]

| **keyword, name** | **probes** | **genes** | **KEGG pathway name** | **probes** | **genes** | **WIKI pathway name** | **probes** | **genes** |
| --- | --- | --- | --- | --- | --- | --- | --- | --- |
| *Antimicrobial peptides, amp* | 22 | **9** | *Antigen processing and presentation* | 133 | **23** | *Alpha6-beta4 integrin signaling* | 51 | **36** |
| *Apoptosis, apo* | 114 | **36** | *Apoptosis* | 206 | **25** | *Apoptosis wikipathway* | 66 | **49** |
| ***Complement,com*** | 107 | **36** | *Bacterial invasion of epithelial cells* | 147 | **35** | *Apoptosis modulation by hsp70* | 19 | **13** |
| *Cluster differentiation antigens,cdi* | 904 | **281** | *B cell receptor signaling pathway* | 117 | **42** | *B-cell receptor wikipathway* | 130 | **94** |
| *Chemokines,chk* | 269 | **45** | *Chemokine signaling pathway* | 155 | **46** | *EGFR1 signaling pathway* | 151 | **103** |
| *Cytochrome, cyp* | 62 | **51** | ***Complement and coagulation cascades*** | 168 | **51** | *EPO receptor signaling* | 25 | **20** |
| *High mobility proteins, hmg* | 35 | **11** | *Cytosolic DNA-sensing pathway* | 90 | **24** | *Erk1-erk2 MAPK cascade* | 151 | **71** |
| *Homeo domain proteins, hom* | 185 | **59** | *Epithelial cell Helicobacter pylori* | 121 | **31** | *Fas pathway and stress induction* | 38 | **28** |
| *Heat shock proteins,hsp* | 444 | **97** | *Fc epsilon RI signaling pathway* | 95 | **59** | *FGF signaling pathway* | 139 | **62** |
| ***Interferon, ifn*** | 116 | **36** | *Fc gamma R-mediated phagocytosis* | 121 | **38** | *G protein signaling pathways* | 70 | **17** |
| *Immunoglobulins, igs* | 159 | **45** | *Hematopoietic cell lineage* | 279 | **58** | *Interleukin2* | 64 | **50** |
| *Interleukins, ils* | 165 | **47** | *Hepatitis C* | 208 | **59** | *Interleukin3* | 88 | **65** |
| *Kinases, kin* | 151 | **60** | *Herpes simplex infection* | 314 | **85** | *Interleukin4* | 46 | **38** |
| *Macrophages, mac* | 105 | **37** | *HTLV-I infection* | 415 | **104** | *Interleukin5* | 57 | **43** |
| ***Major histocompatibility complex, mhc*** | 149 | **34** | *Intestinal immune network IgA* | 150 | **29** | *Interleukin6* | 89 | **61** |
| ***Myxovirus-induced proteins, mx*** | 17 | **6** | *Influenza A* | 318 | **86** | *Interleukin7* | 38 | **29** |
| ***Novel immune-type receptors, nitr*** | 129 | **17** | *Jak-STAT signaling pathway* | 83 | **23** | *Interleukin9* | 19 | **14** |
| *Oncogenes* | 162 | **45** | *Malaria* | 108 | **34** | *Integrin-mediated cell adhesion* | 72 | **49** |
| *T cell receptor, tcr* | 13 | **2** | *MAPK signaling pathway* | 468 | **99** | *MAPK cascade* | 31 | **20** |
| *Toll-like receptors, tlr* | 50 | **21** | *Measles* | 263 | **65** | *MAPK signaling wikipathway* | 136 | **36** |
| *Tumor necrosis factor, tnf* | 97 | **26** | *Natural killer cell mediated cytotoxicity* | 228 | **59** | *P38 MAPK signaling pathway* | 33 | **27** |
| *Transcription factors, tra* | 2300 | **606** | *NOD-like receptor signaling pathway* | 161 | **38** | ***Proteasome degradation*** | 41 | **36** |
| *VHSV-induced proteins, vig* | 20 | **2** | *RIG-I-like receptor signaling pathway* | 138 | **42** | *Senescence and autophagia* | 47 | **42** |
| *Zin finger factors, zin* | 165 | **36** | *T cell receptor signaling pathway* | 168 | **60** | *Signaling of hepatocyte growth* | 36 | **26** |
| ***C-reactive protein, crp*** | 7 | **7** | *TGF-beta signaling pathway* | 188 | **39** | *T-cell receptor wikipathway* | 99 | **71** |
|  |  |  | *Toll-like receptor signaling pathway* | 257 | **60** | *TGFb receptor wikipathway* | 135 | **93** |
|  |  |  | *NF-kappa B signaling pathway* | 239 | **69** | *TGFb signaling wikipathway* | 44 | **26** |
|  |  |  | *PI3K-Akt signaling pathway* | 274 | **73** | *TNFa NFkB signaling* | 154 | **111** |
|  |  |  | *Protein export* | 77 | **19** | *Toll-like receptor wikipathway* | 66 | **53** |
|  |  |  | *Protein processing in endoplasmic reticulum* | 333 | **63** | ***Type II interferon signaling (IFNG)*** | 22 | **19** |
|  |  |  | *Ubiquitin-mediated proteolysis* | 409 | **55** |  |  |  |
|  |  |  | *SNARE interactions vesicular transport* | 64 | **11** |  |  |  |

**S1 Table. Gene Sets (GS) selected in the in-house-designed microarray targeted to zebrafish immune-related genes (Agilent's ID 47562).**

***Bold red***, Top enriched Gene Sets (GS) by GSEA of Table 1 (gene composition in Table S4). Gene composition of all GS in GEOs platform GPL17670.
