# Supplementary material for: Innate Multigene Family Memories Are Implicated in the Viral-Survivor Zebrafish Phenotype
Source: PLoS One. 2015 Aug 13;10(8):e0135483. doi: 10.1371/journal.pone.0135483 (PMC4535885; doi:10.1371/journal.pone.0135483)
Supplement: S2 Table — RTqPCR was used to validate some microarray results using selected differentially expressed genes and to evaluate VHSV replication levels by NVHSV (see methods). Forward and reverse primers amplifying 100–120 bp were designed using the Array Designer 4.3 program (Premier Biosoft Palo Alto CA, USA). The rplp0 gene was used as normalizer gene. (DOCX) [file pone.0135483.s006.docx]

**S2 Table. List of primers used for RTqPCR**

| **gene** |  | **forward** | **reverse** |
| --- | --- | --- | --- |
| ***mxa*** | BC117638.1 | TGGAGCAGGTGTTGGTATCAG | AGGTCAGGAACATTGGCAGAG |
| ***mxb*** | AJ544824.2 | TGGAGCAGGTGTTGGTATCAG | AGGTCAGGAACATTGGCAGAG |
| ***mxc*** | AJ544825.1 | TGGACACTCTCAGGGCATTAG | GCAATAGCAGGCAGGTTCAG |
| ***mxe*** | AJ544827.1 | TGGCTGGAATAGGCGAAGG | TGAGAGTGAGGTCAGGAACATC |
| ***mxg*** | AJ544829.2 | TCAGCAGAAGGCGTAGAGAC | AATACTCAGGCGGTCCAGAC |
| ***mxd*** | AJ544826.1 | TGCGACCTTACACTGATTGATC | CATCTTCTGGTTGTCCTGCTAC |
| ***mxf*** | AJ544828.1 | TTGTGCGGTGTCGTGGTC | TGGTGGCTCCTGAAGAAGTTC |
| ***crp1*** | XM_693995.4 | CCGCCTCTGTCCACCTTC | CGTCCGTCCACCCAGAAG |
| ***crp2*** | BC097160 | AGTCAGACCTGGAGCAAGATG | GCAGCCGCTAATGTCACAG |
| ***crp3*** | BC154042 | GGAGCTGCTGGTCGATAGG | CAACTTCAGATGTGCGGTAGG |
| ***crp4*** | BC115188 | CTCCCTCCTCTCTCCATCTTTC | ACTGCGGCGTCCATTCAC |
| ***crp5*** | BC121777 | ACCTGCTGAATCATACTTGGAG | CCTTCGGTAGCCTCTAATGTC |
| ***crp6*** | BC162745 | TCGCCTACCGCACTTCTG | TTTCCGTCTCCACACATTGAG |
| ***crp7*** | BC150371 | CTGTTCGCCTATCGCACTTC | TGCTCCATCGCTACTAGACTG |
| ***igm*** | BC154613.1 | GCACAATAAGCGGAATGATAGC | GGGTGGGAGGGATGATGTC |
| ***fas*** | XM_685355.3 | TGGTGCCACTCATCCTAATAGC | CTCGGGCGGACCTGAAAG |
| ***ifih*** | XM_689032.2 | GGAGAGGAAGCAGCGGATG | TTGTTGACCAGGACGACCAC |
| ***hsp90*** | NM_001045073 | GAAGAAGCAGGAGGAGAAGAAG | ACGGTTGGAGACGGTGAC |
| ***hsbp1*** | NM_001008615 | CTGTGATGCCTCCTATGATGAC | TGCTTGACCTCCGACATCC |
| ***il1b*** | NM_212844.1 | AGCCTGTGTGTTTGGGAATC | CTTGAGTACGAGATGTGGAGAC |
| ***Nvhsv*** | AJ233396 | GCGTTGTCCGTGCTTCTC | TCCTCCTGTGTGTTCCCTTG |
| ***rplp0*** | NM_131580.2 | CACGCTGCTGAACATGCTGAAC | AATCCTCCTTGGGTGCCTCCTC |

RTqPCR was used to validate microarray results using selected differentially expressed genes and to evaluate *VHSV* replication levels by N*_VHSV_* (see methods). Forward and reverse primers amplifying 100-120 bp were designed by using the Array Designer 4.3 program (Premier Biosoft Palo Alto CA, USA). The *rplp0* gene was used as the normalizer gene.
