# Supplementary material for: Innate Multigene Family Memories Are Implicated in the Viral-Survivor Zebrafish Phenotype
Source: PLoS One. 2015 Aug 13;10(8):e0135483. doi: 10.1371/journal.pone.0135483 (PMC4535885; doi:10.1371/journal.pone.0135483)
Supplement: S3 Table — The list of unique genes with their corresponding normalized mean fluorescent values from 4 biological replicas of pooled head kidney + spleens from 3 zebrafish per replica per phenotype, were used for GSEA. GSEA was performed using the 10295 human GS from its web (msigdb.v4.0.symbols.gmt). GS Enrichment Scores (ES) were normalized for their number of genes (NES) and their False Discovery Rates (FDR) significance assessed by using 1000 gene permutations to estimate null distributions. Only the data with FDR < 0.05 were tabulated and ordered from the highest to the lowest NES. Only 2594 human GS passed the human/zebrafish symbol filter and resulted in the identification of enriched GS. + positive, NES that correlate with the first phenotype in the comparison.—negative, NES that correlate with NI in the comparison. The rest of GSs did not show significant NES. red bold, proteasome/antigen presentation-related GS. Italics, GS related to cell proliferation. Green bold, Apoptosis regulation. Blue bold, interferon-related. Black bold, complement and coagulation cascades. (DOCX) [file pone.0135483.s007.docx]

**S3 Table.** **Significant NES obtained by using GSEA of human GSs from the GSEA data base**

| **VHSV** | **nºgenes** | **NES** |
| --- | --- | --- |
| ***ENK_UV_RESPONSE_KERATINOCYTE_UP*** | ***51*** | ***-1.91*** |
| ***REACTOME_DOWNSTREAM_SIGNALING_EVENTS_OF_B_CELL_RECEPTOR_BCR*** | ***44*** | ***-1.94*** |
| ***REACTOME_CELL_CYCLE*** | ***53*** | ***-2.06*** |
| PID_IL6_7PATHWAY | 21 | -2.10 |
| **PELLICCIOTTA_HDAC_IN_ANTIGEN_PRESENTATION_UP** | **17** | **-2.19** |
| REACTOME_ACTIVATION_OF_NF_KAPPAB_IN_B_CELLS | 35 | -2.21 |
| **WONG_PROTEASOME_GENE_MODULE** | **18** | **-2.33** |
| ***REACTOME_CELL_CYCLE_MITOTIC*** | ***43*** | ***-2.35*** |
| ***REACTOME_S_PHASE*** | ***36*** | ***-2.38*** |
| ***REACTOME_MITOTIC_G1_G1_S_PHASES*** | ***34*** | ***-2.42*** |
| **REACTOME_REGULATION_OF_APOPTOSIS** | 31 | -2.43 |
| REACTOME_P53_DEPENDENT_G1_DNA_DAMAGE_RESPONSE | 32 | -2.51 |
| ***REACTOME_CYCLIN_E_ASSOCIATED_EVENTS_DURING_G1_S_TRANSITION_*** | ***31*** | ***-2.55*** |
| ***REACTOME_G1_S_TRANSITION*** | ***33*** | ***-2.55*** |
| **REACTOME_AUTODEGRADATION_OF_THE_E3_UBIQUITIN_LIGASE_COP1** | **31** | **-2.56** |
| **BIOCARTA_PROTEASOME_PATHWAY** | **16** | **-2.63** |
| ***REACTOME_CELL_CYCLE_CHECKPOINTS*** | ***39*** | ***-2.64*** |
| **REACTOME_CLASS_I_MHC_MEDIATED_ANTIGEN_PROCESSING_PRESENTATION** | **55** | **-2.7** |
| REACTOME_METABOLISM_OF_MRNA | 35 | -2.71 |
| **PROTEASOME_COMPLEX** | **15** | **-2.73** |
| REACTOME_HIV_INFECTION | 43 | -2.75 |
| ***REACTOME_DNA_REPLICATION*** | ***35*** | ***-2.83*** |
| **REACTOME_ANTIGEN_PROCESSING_CROSS_PRESENTATION** | **34** | **-2.84** |
| **REACTOME_ANTIGEN_PROCESSING_UBIQUITINATION_PROTEASOME_DEGRADATION** | **47** | **-2.91** |
| ***REACTOME_P53_INDEPENDENT_G1_S_DNA_DAMAGE_CHECKPOINT*** | ***29*** | ***-2.91*** |
| REACTOME_REGULATION_OF_MRNA_STABILITY_BY_PROTEINS_THAT_BIND_AU_RICH_ELEMENTS | 31 | -2.92 |
| **REACTOME_ER_PHAGOSOME_PATHWAY** | **31** | **-2.92** |
| ***REACTOME_SYNTHESIS_OF_DNA*** | ***32*** | ***-2.92*** |
| REACTOME_AUTODEGRADATION_OF_CDH1_BY_CDH1_APC_C | 31 | -2.94 |
| REACTOME_METABOLISM_OF_RNA | 37 | -2.95 |
| ***REACTOME_MITOTIC_M_M_G1_PHASES*** | ***31*** | ***-2.98*** |
| REACTOME_HOST_INTERACTIONS_OF_HIV_FACTORS | 38 | -2.99 |
| **KEGG_PROTEASOME** | **27** | **-3.00** |
| ***REACTOME_REGULATION_OF_MITOTIC_CELL_CYCLE*** | ***33*** | ***-3.02*** |
| REACTOME_DESTABILIZATION_OF_MRNA_BY_AUF1_HNRNP_D0 | 28 | -3.05 |
| ***REACTOME_M_G1_TRANSITION*** | 28 | -3.06 |
| REACTOME_SIGNALING_BY_WNT | 30 | -3.07 |
| **REACTOME_CROSS_PRESENTATION_OF_SOLUBLE_EXOGENOUS_ANTIGENS_ENDOSOMES** | **28** | **-3.07** |
| REACTOME_APC_C_CDC20_MEDIATED_DEGRADATION_OF_MITOTIC_PROTEINS | 33 | -3.08 |
| REACTOME_VIF_MEDIATED_DEGRADATION_OF_APOBEC3G | 29 | -3.08 |
| **VHSVS** | **nºgenes** | **NES** |
| ***REACTOME_M_G1_TRANSITION*** | ***28*** | ***1.80*** |
| REACTOME_CDT1_ASSOCIATION_WITH_THE_CDC6_ORC_ORIGIN_COMPLEX | 28 | 1.79 |
| **REACTOME_CROSS_PRESENTATION_OF_SOLUBLE_EXOGENOUS_ANTIGENS_ENDOSOMES** | **28** | **1.79** |
| REACTOME_DESTABILIZATION_OF_MRNA_BY_AUF1_HNRNP_D0 | 28 | 1.79 |
| ***REACTOME_P53_INDEPENDENT_G1_S_DNA_DAMAGE_CHECKPOINT*** | ***29*** | ***1.79*** |
| ***REACTOME_CELL_CYCLE_CHECKPOINTS*** | ***39*** | ***1.77*** |
| REACTOME_P53_DEPENDENT_G1_DNA_DAMAGE_RESPONSE | 32 | 1.77 |
| **REACTOME_REGULATION_OF_APOPTOSIS** | **31** | **1.77** |
| **REACTOME_AUTODEGRADATION_OF_THE_E3_UBIQUITIN_LIGASE_COP1** | **31** | **1.77** |
| **REACTOME_ANTIGEN_PROCESSING_UBIQUITINATION_PROTEASOME_DEGRADATION** | **47** | **1.76** |
| **BIOCARTA_PROTEASOME_PATHWAY** | **16** | **1.75** |
| **KEGG_PROTEASOME** | **27** | **1.74** |
| **REACTOME_INTERFERON_ALPHA_BETA_SIGNALING** | **20** | **-1.74** |
| **REACTOME_INTERFERON_SIGNALING** | **29** | **-1.77** |
| **REACTOME_INTERFERON_GAMMA_SIGNALING** | **19** | **-1.81** |
| **KEGG_COMPLEMENT_AND_COAGULATION_CASCADES** | 32 | -1.90 |
| **PEPTIDASE_ACTIVITY** | **20** | **-2.09** |
| KAECH_NAIVE_VS_DAY15_EFF_CD8_TCELL_DN | 23 | -2.17 |
| POOLA_INVASIVE_BREAST_CANCER_UP | 24 | -2.21 |
| MARKEY_RB1_ACUTE_LOF_UP | 24 | -2.21 |
| **GOLDRATH_ANTIGEN_RESPONSE** | **39** | **-2.27** |
| KAECH_NAIVE_VS_MEMORY_CD8_TCELL_DN | 19 | -2.33 |
| **VHSVS+** | **nºgenes** | **NES** |
| KAECH_NAIVE_VS_MEMORY_CD8_TCELL_DN | 19 | -2.14 |
| **ENDOPEPTIDASE_ACTIVITY** | 16 | -2.20 |
| **KEGG_COMPLEMENT_AND_COAGULATION_CASCADES** | **32** | **-2.44** |
| CAIRO_LIVER_DEVELOPMENT_DN | 24 | -2.48 |
| **PEPTIDASE_ACTIVITY** | **20** | **-2.54** |

The list of unique genes with their corresponding normalized mean fluorescent values from 4 biological replicas of pooled head kidney + spleens from 3 zebrafish per replica per phenotype. were used for GSEA. GSEA was performed using the 10295 human GS from its web (msigdb.v4.0.symbols.gmt). GS Enrichment Scores (ES) were normalized for their number of genes (NES) and their False Discovery Rates (FDR) significance assessed by using 1000 gene permutations to estimate null distributions. Only the data with FDR < 0.05 were tabulated and ordered from the highest to the lowest NES. Only 2594 human GS passed the human/zebrafish symbol filter and resulted in the identification of enriched GS. **+ positive**. NES that correlate with the first phenotype in the comparison. **– negative,** NES that correlate with NI in the comparison. The rest of GS did not show significant NES. **red bold**, proteasome/antigen presentation-related GS. ***Italics***, GS related to cell proliferation. **Green bold**, Apoptosis regulation. **Blue bold**, interferon-related. **Black bold**, complement and coagulation cascades.
