# Supplementary material for: Innate Multigene Family Memories Are Implicated in the Viral-Survivor Zebrafish Phenotype
Source: PLoS One. 2015 Aug 13;10(8):e0135483. doi: 10.1371/journal.pone.0135483 (PMC4535885; doi:10.1371/journal.pone.0135483)
Supplement: S4 Table — Due to the small number of genes in the GSs defined by the crp and mx keywords, other related genes were added to reach the gene number requirements for estimation of significance. (DOCX) [file pone.0135483.s008.docx]

**S4 Table. Gene composition of the top GSs from GSEA of Table 1**

| ***mx*** | ***nitr*** | ***ifn*** | ***Complement***  ***coagulation***  ***cascades*** | ***com*** | ***crp*** | ***mhc*** | ***type II interferon signaling*** | ***proteasome degradation*** |
| --- | --- | --- | --- | --- | --- | --- | --- | --- |
| *mxa* | *nitr10* | *igsf* | *f12* | *c5* | *crp2* | *hla28* | *eif* | *psmc1* |
| *mxb* | *nitr11* | *gig2* | *f9b* | *fib* | *crp4* | *ciita* | *il1b* | *h2afx* |
| *mxe* | *nitr12* | *ifih* | *f13a1* | *b/c2b* | *crp5* | *cr5l* | *stat1a* | *psma8* |
| *mxg* | *nitr13* | *isgf3* | *f2* | *c13orf22* | *crp7* | *mhc3* | *spi1* | *psmb2* |
| *mxc* | *nitr14* | *ifngilrl* | *f10* | *c1qtnf* | *crp3* | *taich* | *socs1* | *psmd8* |
| *mxd* | *nitr1* | *mxa* | *egf* | *c1rs* | *crp6* | *bing* | *cybb* | *psmd13* |
| *mxf* | *nitr2* | *igf2* | *cd248* | *c2orf24* | *crp1* | *daxx* | *ifit2* | *psmc6* |
| *ifn1-2* | *nitr3* | *igf1r* | *f2r* | *clu* | *sap* | *ke6* | *stat2* | *psmb6* |
| *ifnphi1* | *nitr4a* | *ifi30* | *serpind* | *coch* | *sapp* | *pplc* | *ciita* | *psme1* |
| *ifnphi2* | *nitr5* | *ifngr1* | *fga* | *f3* | *c1q* | *tapasin* | *irf1b* | *psmb10* |
| *ifnphi3* | *nitr6* | *ifniptt5l* | *pros1* | *f10* | *c1s* | *hcpl* | *irf2* | *psmb1* |
| *irf6* | *nitr7* | *ifnitml* | *proc* | *c3h* | *serping1* | *hm13* | *nos* | *psmc4* |
| *irf7* | *nitr8* | *ifnphi1* | *a2m* | *c4* | *c2* | *hmha1* | *irf8* | *psmb5* |
| *irf10* | *nir* | *ifnphi2* | *tfpia* | *c1q* | *c4* | *hm2mb2* | *prkc* | *psmd9* |
| *ifih* | *nitr9* | *ifnphi3* | *serpinc* | *c3l* | *vwf* | *hm2q10* | *jak1* | *rpn* |
| *il1b* | *nitr-h* | *irf1* | *vwf* | *c6* | *c3* | *hm2c1* | *irf4* | *psma2* |
| *vig* | *nitr* | *irf10* | *f5* | *c7* | *c1qr* | *bat1* | *ptpn* | *ube2d* |
|  |  | *irf11* | *f3* | *c8* | *serpine-h* | *hladpa* | *irf9* | *psme2* |
|  |  | *irf2* | *kng1* | *c9* | *serpine* | *iclp* | *psmb9* | *psmd2* |
|  |  | *irf2bp* | *klkb1* | *bfb* |  | *mhc1ufa* | *jak2b* | *psmc5* |
|  |  | *irf3* | *plaur* | *c1qr* |  | *mhc1uda* | *ifn1-2* | *psme3* |
|  |  | *irf4* | *cpb2* | *c3* |  | *mhc1uea* | *ifnphi1* | *psmb4* |
|  |  | *irf5* | *plg* | *c3b* |  | *mhc1uxa* | *ifnphi2* | *sb10l* |
|  |  | *irf7* | *serpina* | *c3c* |  | *mhc2a-h* | *ifnphi3* | *psmb3* |
|  |  | *irf8* | *serpinf* | *cfb* |  | *mhc2avc* | *vig* | *psmc3* |
|  |  | *irf9* | *plaub* | *cfdl* |  | *mhc2b* |  | *psmb7* |
|  |  | *ifrd* | *plat* | *cfh* |  | *mhc2b-h* |  | *psmd11b* |
|  |  | *isg20l2* | *serpine-h* | *cfi* |  | *mhc1* |  | *psmd12* |
|  |  | *nsiifnl* | *serpine* | *properdin* |  | *mhc1ze* |  | *psma5* |
|  |  | *prkri* | *bdkrb1* | *crl* |  | *rfx* |  | *psma4* |
|  |  | *isg12* | *cfi* | *H* |  | *mhc1uaa* |  | *uba* |
|  |  | *irf6* | *cfdl* | *prf-h* |  | *mhc1uba* |  | *psmd10* |
|  |  | *trim33* | *c3b* | *prf* |  | *mhc1uca* |  | *psmd3* |
|  |  | *trim* | *cfb* | *serpine* |  | *mhc2a* |  | *psmd7* |
|  |  | *tfdp* | *c3ar1* | *sushi* |  |  |  | *psmc2* |
|  |  | *ifng1-2* | *clr1l* | *cd142* |  |  |  | *psmb8* |
|  |  |  | *cd59* |  |  |  |  |  |
|  |  |  | *c8* |  |  |  |  |  |
|  |  |  | *c7* |  |  |  |  |  |
|  |  |  | *c6* |  |  |  |  |  |
|  |  |  | *c5* |  |  |  |  |  |
|  |  |  | *serping* |  |  |  |  |  |
|  |  |  | *c1s* |  |  |  |  |  |
|  |  |  | *c1q* |  |  |  |  |  |
|  |  |  | *c4* |  |  |  |  |  |
|  |  |  | *c2* |  |  |  |  |  |
|  |  |  | *hbl* |  |  |  |  |  |
|  |  |  | *masp* |  |  |  |  |  |
|  |  |  | *cr2* |  |  |  |  |  |
|  |  |  | *cd55* |  |  |  |  |  |

Due to the small number of genes in the GSs defined by the *crp* and *mx* keywords, other related genes were added to reach the gene number requirements for estimation of significance
