# Supplementary material for: Innate Multigene Family Memories Are Implicated in the Viral-Survivor Zebrafish Phenotype
Source: PLoS One. 2015 Aug 13;10(8):e0135483. doi: 10.1371/journal.pone.0135483 (PMC4535885; doi:10.1371/journal.pone.0135483)
Supplement: S5 Table — Red, significantly enriched novel GSs (Table 2). (DOCX) [file pone.0135483.s009.docx]

**S5 Table. Gene composition of novel GSs proposed by clustering the Leading Edge enriched genes according to the GSEA results of Table 1**

| ***9cxcs*** | ***5ifn***  ***+4mx*** | ***8tlr+7ifn***  ***+5mx*** | ***7mapks*** | ***1ig***  ***3mapk*** | ***7oncos*** | ***1creb*** | ***278casp*** | ***2348tlr***  ***+12ifn*** | ***1nfkb***  ***2nfkbiab*** | ***7tlr***  ***7casp*** | ***23789***  ***casps*** | ***12mapks***  ***+5pirp*** | ***mapk***  ***+pik*** |
| --- | --- | --- | --- | --- | --- | --- | --- | --- | --- | --- | --- | --- | --- |
| *bcar* | ***aicda*** | ***aicda*** | *araf* | *akt3a* | *abi1b* | *atp2b4* | *actl6a* | *akt2* | *apaf1* | *ahsa1* | ***abi1b*** | ***abi1b*** | *acp1* |
| *bcl2* | ***caspa*** | ***azi2*** | *atf* | *asap2* | *ahsa* | *bcl2* | *apaf1* | *akt3* | *araf* | *canx* | ***abl*** | ***acp1*** | *akt2* |
| *cc19l* | ***ciita*** | ***blk*** | *braf* | *ccnd1* | *araf* | *blk* | *azi2* | *atm* | *bdnf* | *casp7* | ***akt3a*** | ***akt2*** | *akt3* |
| *ccl4l* | ***furinb*** | ***cc*** | *ek1* | *cish* | *cebpb* | *ccnd2a* | *bag* | *bad* | *braf* | *ccl25b* | ***apaf1*** | ***akt3*** | *arhgdig* |
| *cclc* | ***ifih*** | ***cc21l*** | *fgf* | *crk* | *chek* | *cdk* | *bcl* | *bax* | *casp2* | *cd276* | ***atf*** | ***alox5*** | *braf* |
| *cd169* | ***ifng1-2*** | ***ccl2*** | *igf2* | *fancl* | *cpla2* | *cmtm3* | *casp2* | *bida* | *cc21l* | *cxcl* | ***badb*** | ***alpo*** | *casp9* |
| *cd22* | ***ifnphi1*** | ***ccl4l*** | *kdr* | *ighmc* | *crya* | *creb* | *casp7* | *ccl5* | *ccl5* | *egfra* | ***bax*** | ***appl2*** | *cblb* |
| *cd53* | ***ikbke*** | ***ccl5*** | *map2k6* | *map2k1* | *daxx* | *dvl1b* | *casp8* | *fas* | *cdc25* | *ifi30* | ***bbc3*** | ***arhgdig*** | *cd53* |
| *crfa* | ***il1b*** | ***chuk*** | *map3k7* | *mapk1* | *eef* | *e2f* | *cav* | *faslg* | *cflar* | *ikbke* | ***bcl2*** | ***arpc*** | *cdk5r1b* |
| *cxc46* | ***irak3*** | ***cmoti*** | *map4k* | *marcks* | *egf* | *gps2* | *cflar* | *fcgr3a* | *chuk* | *il1b* | ***becn1*** | ***atp2b4*** | *csf1a* |
| *cxc64* | ***irf10*** | ***cxcl*** | *mapk14* | *mcl1* | *elk* | *hnrpkl* | *chuk* | *fk* | *dhx* | *irf6* | ***blp1*** | ***azi2*** | *dcun1d5* |
| *cxcl* | ***irf7*** | ***dhx*** | *mapk4* | *plcg* | *eps15* | *htatip* | *cops* | *furinb* | *eif3* | *lgmn* | ***bnip*** | ***braf*** | *eif* |
| *cxxr3.1* | ***mhc1uba*** | ***fbxw11*** | *mapk6* | *prkc* | *fgf* | *lck* | *cul* | *ifih* | *gadd45* | *map3k6* | ***capn*** | ***cbl*** | *elk* |
| *dapp1* | ***mhc1uea*** | ***ifih*** | *mapkap* | *ptk2.1* | *fos* | *mad* | *cylda* | *ifng1-2* | *git1l* | *myd88* | ***casp2*** | ***cblb*** | *epn1* |
| *gab* | ***mxa*** | ***ifnphi1*** | *max* | *pxn* | *frs2b* | *map3k2* | *faf1* | *ifnphi3* | *grb* | *nf1b* | ***casp3*** | ***cd22*** | *fynb* |
| *inpp5d* | ***mxb*** | ***igf1r*** | *mknk* | *rac1* | *gsc* | *mbpb* | *fancd2* | *il10* | *jund* | *pltp* | ***casp7*** | ***crk*** | *git2a* |
| *itm* | ***mxc*** | ***ikbkb*** | *mycl* | *rps6k* | *hip1* | *nfy* | *ikbke* | *il12* | *lamtor3* | *stm* | ***casp8*** | ***crmp1*** | *gsk3* |
| *jak1* | ***mxe*** | ***irf6*** | *ptenb* |  | *hsf* | *pdgfr* | *ikbkg* | *il1b* | *map2k6* | *tirap* | ***casp9*** | ***cth1*** | *homer3* |
| *jak3* | ***nlrp*** | ***irf7*** | *raf1* |  | *hsp90* | *phd* | *irf9* | *irak1* | *mapk3* | *tlr7* | ***caspas*** | ***dnm*** | *il6r* |
| *pip4k2a* | ***pycard*** | ***isg12*** | *rps6* |  | *hspb* | *pip4k2a* | *map3k3* | *irak3* | *mapk7* | *tnfr* | ***caspb*** | ***elk*** | *il6st* |
| *ppp3r* | ***rsad2*** | ***isg15*** | *serpine* |  | *jak1* | *plcb3* | *mcm* | *irak4* | *mapkap* | *vdccg3* | ***caspc*** | ***gab*** | *inppl1* |
| *ptpn* | ***vig*** | ***mxa*** | *sh3g* |  | *jak2b* | *ppp3* | *mtif2* | *irf10* | *mos* |  | ***ccnd1*** | ***git1l*** | *itgb* |
| *ras* |  | ***mxb*** | *tradd* |  | *jun* | *prex1* | *nlrx1* | *irf6* | *nfkb2* |  | ***cdc25*** | ***git2a*** | *lpar* |
| *rasgrp* |  | ***mxc*** |  |  | *jund* | *vcam1* | *otud5a* | *irf7* | *nfkbiab* |  | ***cdk5r1b*** | ***homer3*** | *map2k1* |
| *sicya* |  | ***mxe*** |  |  | *map2k2* | *zfp* | *parp1* | *ivns1abp* | *nr4* |  | ***cflar*** | ***il10*** | *map2k5* |
| *sicyb* |  | ***mxg*** |  |  | *map3k5* |  | *pebp1* | *kir2ds1* | *pak2a* |  | ***col17a1*** | ***lfg*** | *mapk12a* |
| *stat1a* |  | ***nlrp*** |  |  | *mapk15* |  | *pik3* | *klrc2* | *parp1* |  | ***cradd*** | ***map2k5*** | *mapk4* |
| *stat3* |  | ***nsiifnl*** |  |  | *mapk7* |  | *rasgab* | *klrd1* | *pidd* |  | ***daxx*** | ***map3k2*** | *mapk6* |
| *syk* |  | ***otud5a*** |  |  | *mcf2a* |  | *rel* | *litaf* | *pin1* |  | ***egfra*** | ***map3k3*** | *mapk7* |
| *tec* |  | ***phd*** |  |  | *mink1* |  | *smarc* | *map3k7* | *psme3* |  | ***eps15*** | ***mapk1*** | *mtor* |
| *xcr1a* |  | ***pidd*** |  |  | *myc* |  | *sumo* | *mapk10* | *rap1* |  | ***erbb*** | ***mapk12a*** | *pak4* |
|  |  | ***plaub*** |  |  | *mycn* |  | *trpc4ap* | *mapk11* | *rasa* |  | ***errfi1*** | ***mapk14*** | *pdpk1* |
|  |  | ***polr3f*** |  |  | *nlk2* |  | *txlna* | *mapk12a* | *stk* |  | ***fadd*** | ***mapk3*** | *pik3a* |
|  |  | ***pscapl*** |  |  | *nr2* |  | *ube* | *mapk9* | *tcirg1* |  | ***faf1*** | ***mapk4*** | *pik3c* |
|  |  | ***ptgs2b*** |  |  | *nr4* |  |  | *msna* | *tert* |  | ***fas*** | ***mapk6*** | *pkn* |
|  |  | ***pycard*** |  |  | *nup* |  |  | *mxa* | *tpte* |  | ***faslg*** | ***mapk7*** | *ppp2ca* |
|  |  | ***rel*** |  |  | *pcdh8* |  |  | *nlrp* | *tradd* |  | ***flna*** | ***mapk9*** | *prkd* |
|  |  | ***rhobtb2*** |  |  | *pdgfa* |  |  | *pik3a* | *trim* |  | ***foxo*** | ***marcks*** | *pstpip1a* |
|  |  | ***rsad2*** |  |  | *pi3ka* |  |  | *relt* | *xiap* |  | ***git1l*** | ***nck*** | *ptenb* |
|  |  | ***sgut1*** |  |  | *pla2* |  |  | *tab* |  |  | ***git2a*** | ***otud5a*** | *ptk2bb* |
|  |  | ***sicya*** |  |  | *ppp3r* |  |  | *tlr2* |  |  | ***gnb*** | ***pac4*** | *ptprja* |
|  |  | ***spred1*** |  |  | *ppsc* |  |  | *tlr3* |  |  | ***grk7a*** | ***pak4*** | *rac2* |
|  |  | ***tirap*** |  |  | *ptk2bb* |  |  | *tlr4* |  |  | ***gsk3*** | ***pdk*** | *rap1* |
|  |  | ***tlr21*** |  |  | *ras* |  |  | *tlr8* |  |  | ***hdac*** | ***pebp1*** | *rapgef* |
|  |  | ***tlr22*** |  |  | *rasgrp* |  |  | *tnfa* |  |  | ***hnf4a*** | ***phd*** | *rheb* |
|  |  | ***tlr3*** |  |  | *rgs5b* |  |  | *tnfaip* |  |  | ***hsp90*** | ***pik3a*** | *rps6* |
|  |  | ***tlr4*** |  |  | *shcbp* |  |  | *tnfb* |  |  | ***htt*** | ***pik3c*** | *shc* |
|  |  | ***tlr5*** |  |  | *sos1* |  |  | *tnfsf* |  |  | ***igf1*** | ***pik3r5*** | *src* |
|  |  | ***tlr7*** |  |  | *srf* |  |  | *traf3ip1* |  |  | ***il3*** | ***pip5k*** | *stppp* |
|  |  | ***tlr8*** |  |  | *stat3* |  |  | *traf5* |  |  | ***il6r*** | ***pl10*** | *them4* |
|  |  | ***tlr9*** |  |  | *stk* |  |  | *trim* |  |  | ***il6st*** | ***pla2g*** | *tsc* |
|  |  | ***tmem*** |  |  | *stpktao* |  |  | *xpo1b* |  |  | ***ing1*** | ***pld1a*** | *tub* |
|  |  | ***tolloid*** |  |  | *stxbp1* |  |  |  |  |  | ***inppl1*** | ***ppp3r*** | *vasp* |
|  |  | ***traf6*** |  |  | *usf* |  |  |  |  |  | ***irf2*** | ***prkc*** |  |
|  |  | ***trim*** |  |  | *vegfaa* |  |  |  |  |  | ***irf3*** | ***prkd*** |  |
|  |  | ***vig*** |  |  | *wasla* |  |  |  |  |  | ***krt*** | ***pstpip1a*** |  |
|  |  |  |  |  |  |  |  |  |  |  |  |  |  |

**Bold red**, significant enriched novel GSs (Table 2). Additional genes included in the ***23789****casps* GS were: ***lama, lamb1, lamp2, ldlr, lta, map1lc3a, map2k4, map2k5, map2k7, map3k7, mapk4, mapk8, mapk8ip, mapk9, mapkapk, marcks, max, mecom, mink1, mt, nf1b, ngf, nlk2, nod1, ntn1a, oclna, otr, pak1, pard3, phoxh, pkc, pla2g, plcg, pparab, prka, ptk2bb, ptprz1, raf1, rap1ga, rasgrp, rb1, ripk, runx, sfrp, skiipl, smad, smad3, snx6, sphk2, spna2, stam, stat1a, stat2, stat3, stppp, tec, tp53, tp73, traf5, trail, trap1, vdr, wasf2, yes1, yrk, zak.*** Additional genes included in the ***12mapks+5pirp*** GS **were: *ptprja, rac1, rac2, rap1, rap1ga, rapgef, ras, rasgrp, res1, shc, src, tub, vasp, wasf2.***
