# Supplementary material for: Innate Multigene Family Memories Are Implicated in the Viral-Survivor Zebrafish Phenotype
Source: PLoS One. 2015 Aug 13;10(8):e0135483. doi: 10.1371/journal.pone.0135483 (PMC4535885; doi:10.1371/journal.pone.0135483)
Supplement: S6 Table — Membrane, activating and secreting genes, were selected to design cell GSs from different sources. The selected genes were then filtered by its presence on the in-house microarray and the resulting gene lists were used as input for GSEA. Th1, T helper 1 cells. Th2, T helper 2 cells. Th17, T helper 17 cells. Treg, T regulatory cells. B, IgM producing cells. BZ, IgZ producing cells. Dendritic, dendritic cells. Cytotoxic, antigen-specific cytotoxyc cells. NK, natural killer cells. Macrophages, monocyte and macrophages. Neutrophil, neutrophil and granulocyte cells. (DOCX) [file pone.0135483.s010.docx]

**S6 Table. Gene composition of the GSs defining immune cell markers**

| **Th1** | **Th2** | **Th17** | **Treg** | **B** | **BZ** | **dendritic** | **cytotoxic** | **NKcells** | **macrophages** | **neutrophil** |
| --- | --- | --- | --- | --- | --- | --- | --- | --- | --- | --- |
| *ccr* | *ccr* | *ccr* | *cd1* | *cd22* | *cd22* | *cd209* | *cd2* | *cd226* | *anpep* | *anpep* |
| *cd1* | *cd1* | *cd1* | *cd2* | *cd38* | *cd38* | *cd45* | *cd2* | *cd3* | *cd169* | *cd4* |
| *cd2* | *cd2* | *cd2* | *cd3* | *cd40* | *cd40* | *cd83* | *cd3* | *cd38* | *cd209* | *cd45* |
| *cd3* | *cd3* | *cd3* | *cd38* | *cd5* | *cd5* | *csf1a* | *cd8* | *cd4* | *cd45* | *cd45* |
| *cd38* | *cd38* | *cd38* | *cd4* | *cd79* | *cd79* | *ifnphi1* | *eomes* | *cd44* | *cd64* | *csf2* |
| *cd4* | *cd4* | *cd4* | *cd44* | *cd82* | *cd82* | *ifnphi2* | *fasl* | *cd45* | *csf1ra* | *csfr* |
| *cd44* | *cd44* | *cd44* | *cd45* | *cd9* | *cd9* | *ifnphi3* | *fasl* | *cd8* | *ifngr1* | *defb1* |
| *cd45* | *cd45* | *cd45* | *cd5* | *dntt* | *dntt* | *il12* | *gzmb* | *cd8* | *ifnphi1* | *defb1* |
| *cd5* | *cd5* | *cd5* | *cd7* | *fcer2* | *fcer2* | *il4* | *gzmb* | *csf1a* | *ifnphi2* | *defb2* |
| *cd7* | *cd7* | *cd7* | *ctla4* | *ifng1-2* | *ifng1-2* | *mhc2dab* | *icam* | *cxcr* | *ifnphi3* | *defb3* |
| *csf1a* | *cxcr* | *defb1* | *dntt* | *igmh* | *ighz1* | *mhc2dfb* | *il10* | *cxcr4* | *il10* | *il11b* |
| *cxcr* | *dntt* | *defb2* | *il10* | *il10* | *il10* |  | *prf* | *cxcr4* | *il18* | *il13ra* |
| *dntt* | *gata* | *defb3* | *il2* | *il2* | *il2* |  | *tbx* | *cxcr7* | *il1b* | *il1b* |
| *gata* | *ifng1-2* | *dntt* | *il2r* | *il4* | *il4* |  | *tcra* | *fcgr3a* | *il1b* | *il6* |
| *icam1* | *ifngr1* | *icos* | *il7r* | *il5* | *il5* |  | *thy1* | *gzmb* | *il21r* | *il6r* |
| *ifng1-2* | *il10* | *ifng1-2* | *kitlga* | *il6* | *il6* |  | *tnfa* | *il10* | *il3* | *itga* |
| *ifngr1* | *il10* | *il17* | *lrrc* | *il7* | *il7* |  | *tnfb* | *il12* | *il4* | *kitlga* |
| *ifngr1* | *il13* | *il1b* | *smad2* | *il7r* | *il7r* |  |  | *il12* | *il6* | *kitlga* |
| *il10* | *il17* | *il2* | *smad3* | *mhc2dab* | *mhc2dab* |  |  | *il12r* | *il6r* | *mag* |
| *il12r* | *il1r* | *il21* | *stat5* | *mhc2dfb* | *mhc2dfb* |  |  | *il15* | *il6r* | *siglet* |
| *il12r* | *il2* | *il21r* | *stat5* | *mmel1* | *mmel1* |  |  | *il18* | *il8* | *sos2* |
| *il18r* | *il21* | *il22* | *tcra* | *ms4a17* | *ms4a17* |  |  | *il2* | *itga* |  |
| *il2* | *il33* | *il23r* | *tfr* | *tgfb* | *tgfb* |  |  | *il7* | *mag* |  |
| *il2r* | *il4* | *il23r* | *tgfb* | *tgfb2* | *tgfb2* |  |  | *irf1* | *mhc2dab* |  |
| *il4* | *il4r* | *il26* | *tgfb2* | *tgfb3* | *tgfb3* |  |  | *kir2ds1* | *mhc2dfb* |  |
| *il7r* | *il5* | *il4* | *tgfb3* | *vwf* | *vwf* |  |  | *ncam* | *nos* |  |
| *lfa1* | *il7r* | *il6* | *tnfr* |  |  |  |  | *nitr* | *sos2* |  |
| *obscn* | *irf4* | *il6r* | *tnfr* |  |  |  |  | *nitr1* | *tgfb* |  |
| *stat1* | *stat5* | *il7r* | *vwf* |  |  |  |  | *nitr2* | *tgfb2* |  |
| *stat4* | *stat6* | *irf4* |  |  |  |  |  | *nitr2* | *tgfb3* |  |
| *tbx* | *tcra* | *kitlga* |  |  |  |  |  | *nitr3* | *tlr1* |  |
| *tcra* | *tfr* | *rora* |  |  |  |  |  | *nitr4a* | *tlr2* |  |
| *tfr* | *vwf* | *stat3* |  |  |  |  |  | *nitr5* | *tlr4* |  |
| *tnfa* |  | *tcra* |  |  |  |  |  | *nitr6* | *tlr6* |  |
| *tnfa* |  | *tfr* |  |  |  |  |  | *nitr7* | *tnfa* |  |
| *vwf* |  | *tgfb* |  |  |  |  |  | *nitr8* | *tnfb* |  |
|  |  | *tgfb2* |  |  |  |  |  | *nitr9* |  |  |
|  |  | *tgfb3* |  |  |  |  |  | *nkl* |  |  |
|  |  | *tgfr* |  |  |  |  |  | *tcra* |  |  |
|  |  | *tnfa* |  |  |  |  |  | *tgfb* |  |  |
|  |  | *vwf* |  |  |  |  |  | *tgfb2* |  |  |
|  |  |  |  |  |  |  |  | *tgfb3* |  |  |
|  |  |  |  |  |  |  |  | *tnfa* |  |  |

Membrane, activating and secreting genes, were selected to design cell GSs from different sources. The selected genes were then filtered by its presence on the in-house microarray and the resulting gene lists were used as input for GSEA. **Th1**, T helper 1 cells. **Th2**, T helper 2 cells . **Th17**, T helper 17 cells. **Treg,** T regulatory cells. **B,** IgM producing cells. **BZ,** IgZ producing cells. **Dendritic**, dendritic cells. **Cytotoxic,** antigen-specific cytotoxyc cells. **NK**, natural killer cells. **Macrophages**, monocyte and macrophages. **Neutrophil,** neutrophil and granulocyte cells.
